# Experience of early-life pain in premature infants is associated with atypical cerebellar development and later neurodevelopmental deficits

**DOI:** 10.1186/s12916-023-03141-w

**Published:** 2023-11-14

**Authors:** Kevin M. Cook, Josepheen De Asis-Cruz, Jung-Hoon Kim, Sudeepta K. Basu, Nickie Andescavage, Jonathan Murnick, Emma Spoehr, Melissa Liggett, Adré J. du Plessis, Catherine Limperopoulos

**Affiliations:** 1 Developing Brain Institute, Children’s National Hospital, 111 Michigan Ave NW, Washington, DC 20010 USA; 2 Dept. of Diagnostic Imaging & Radiology, Children’s National Hospital, 111 Michigan Ave. NW, Washington, D.C 20010 USA; 3 Division of Psychology, Children’s National Hospital, 111 Michigan Ave. NW, Washington, DC 20010 USA; 4 Prenatal Pediatrics Institute, Children’s National Hospital, 111 Michigan Ave NW, Washington, DC 20010 USA

**Keywords:** Early-life pain, fMRI, Brain development, Cerebellum, Prematurity, Neonatal intensive care unit, Neurodevelopment, Autism spectrum disorder, Language development, Motor development

## Abstract

**Background:**

Infants born very and extremely premature (V/EPT) are at a significantly elevated risk for neurodevelopmental disorders and delays even in the absence of structural brain injuries. These risks may be due to earlier-than-typical exposure to the extrauterine environment, and its bright lights, loud noises, and exposures to painful procedures. Given the relative underdeveloped pain modulatory responses in these infants, frequent pain exposures may confer risk for later deficits.

**Methods:**

Resting-state fMRI scans were collected at term equivalent age from 148 (45% male) infants born V/EPT and 99 infants (56% male) born at term age. Functional connectivity analyses were performed between functional regions correlating connectivity to the number of painful skin break procedures in the NICU, including heel lances, venipunctures, and IV placements. Subsequently, preterm infants returned at 18 months, for neurodevelopmental follow-up and completed assessments for autism risk and general neurodevelopment.

**Results:**

We observed that V/EPT infants exhibit pronounced hyperconnectivity within the cerebellum and between the cerebellum and both limbic and paralimbic regions correlating with the number of skin break procedures. Moreover, skin breaks were strongly associated with autism risk, motor, and language scores at 18 months. Subsample analyses revealed that the same cerebellar connections strongly correlating with breaks at term age were associated with language dysfunction at 18 months.

**Conclusions:**

These results have significant implications for the clinical care of preterm infants undergoing painful exposures during routine NICU care, which typically occurs without anesthesia. Repeated pain exposures appear to have an increasingly detrimental effect on brain development during a critical period, and effects continue to be seen even 18 months later.

**Supplementary Information:**

The online version contains supplementary material available at 10.1186/s12916-023-03141-w.

## Background

Nearly 1 out of 10 babies worldwide are born premature, with minimal change over the past decade [1–4], although survival rates have increased significantly [5, 6]. Despite increasing survival, infants born very preterm (VPT) < 32 weeks postmenstrual age (PMA) and extremely preterm (EPT) < 28 weeks PMA [7] remain at heightened risk for developmental deficits even in the absence of any detectable brain injury, especially sensorimotor [8–10], language [11, 12], and cognitive [10, 12, 13] impairments. Moreover, they experience higher rates of neurodevelopmental disorders such as autism spectrum disorder (ASD) [14–17].

While the association between prematurity and developmental outcomes is well established, mechanisms driving the association remain unclear. One possibility is alterations in brain development arising from earlier-than-typical exposure to the extrauterine environment. The late second through third trimester is a critical period for brain development, seeing rapid increases in global and regional brain volumes [18–22], strengthening of white matter tracts [23, 24], and organizing of functional brain networks [25, 26]. While V/EPT infant’s brains exhibit volumetric increases [27], it is less than term-born infants, especially in the cerebellum and brainstem [22, 28–30]. Furthermore, functional network development in preterm infants is disrupted compared to term-born infants [31, 32].

Importantly, not all infants born V/EPT will experience neurodevelopmental impairments. It is likely the qualities of experiences during this critical period play a role in modifying trajectories. Neonatal intensive care units (NICU) are significantly more sensorially intense relative to the intrauterine environment, with bright lights and loud noises that reach as high as 85 decibels [33, 34]. Exposure to these intense sensory experiences have been linked to altered development (for review see Ream & Lehwald (2018)) [35]. In fact, one study found that the rates of neurodevelopmental disorders such as ASD are higher following a NICU stay even in term-born infants, suggesting NICU exposure itself may play a role [36].

Lengthy NICU stays for V/EPT infants include exposures to various noxious stimuli, especially repeated painful exposures. These infants are regularly exposed to painful procedures over the course of their clinical care, including heel sticks, venipunctures, and catheterizations. Infants born VPT experience an average of 115 painful procedures during their NICU stay, with some reporting over 600 [37] and most occur in the absence of any analgesia [38–40]. Pain pathways are vulnerable to perturbations in V/EPT infants, because of asynchronicity in the earlier developing afferent nociceptive pathways and later maturating efferent modulatory responses [41], suggesting the development of pain sensations precedes the ability to moderate the experience of pain. Research into the effects of painful exposures in the NICU on functional brain development is relatively limited; however, greater NICU pain exposures have been associated with other health and neurodevelopmental alterations including decreased infant weight [42], lower white matter fractional anisotropy, and lower IQ at school age [43], as well as decreased cerebellar volume at school age [44]. Moreover, animal models have linked early pain exposures to increased neuroinflammation and hippocampal development [45], as well as greater neural sensitivity to future noxious exposures in the somatosensory and prefrontal cortex [46]. Most of this research focuses on differences later into childhood and emphasizes structural abnormalities. This results in a significant gap in our understanding of how these experiences change the functional organization of the brain and how early in development these differences can be detected—an essential step in identifying periods of early intervention.

This study aims to examine the association between early pain exposures in both near-term development of functional connections and later neurodevelopment. Using a cohort of V/EPT infants who underwent resting state fMRI at term-equivalent age (TEA), we examined differences in early functional connectivity from term-born infants. We then assessed whether NICU skin break procedures prior to TEA were associated with changes in inter-regional functional brain connectivity. Thereafter, we evaluated whether breaks were associated with risk for neurodevelopmental difficulties at 18 months, and whether the connectivity in regions sensitive to pain were associated with deficits at 18 months.

## Methods

### Participants

**Fig. 1:**
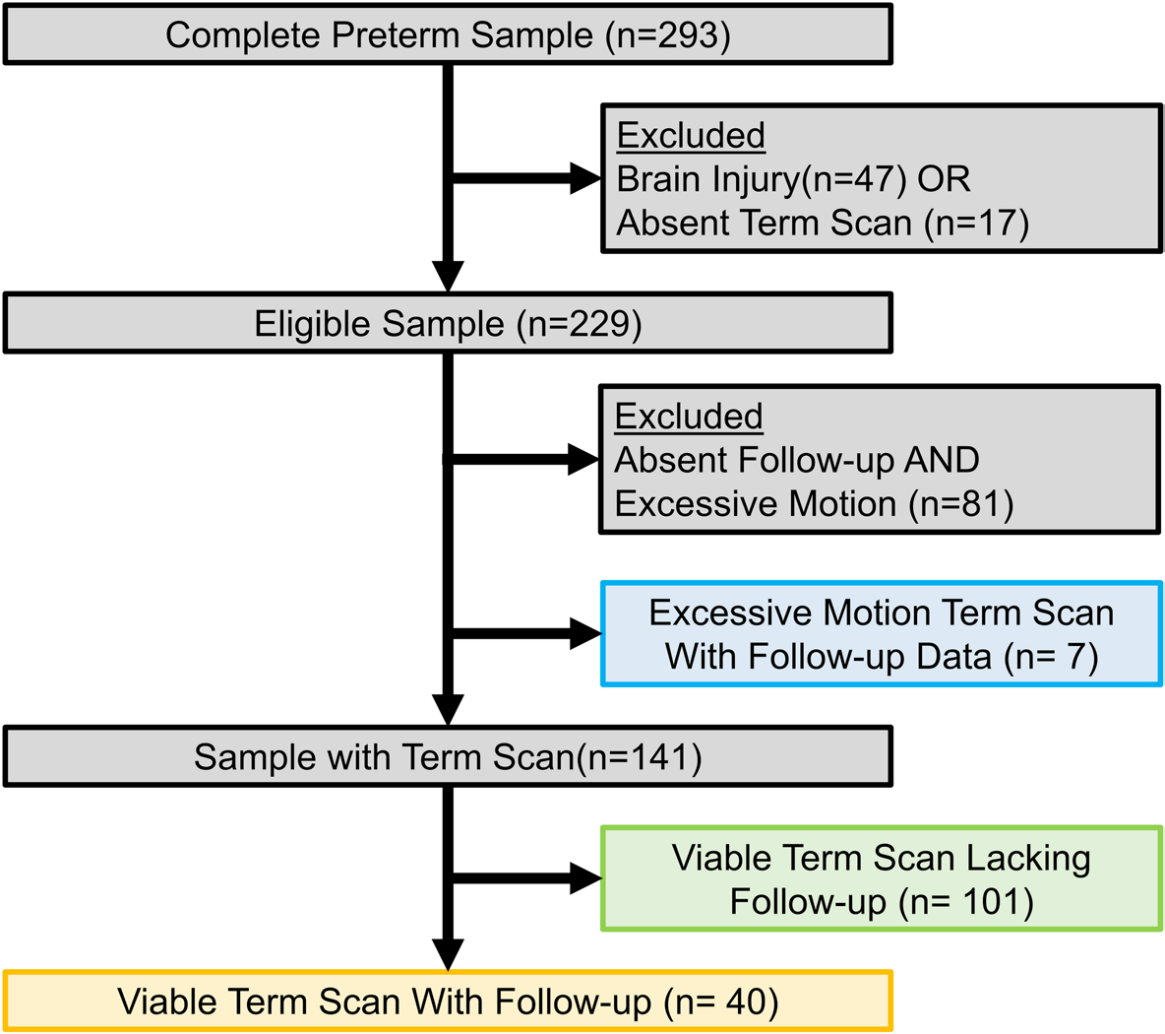
Participant flowsheet. Participant exclusion for the final sample, including all participants with viable term age scans (*n* = 141, green + yellow), participants with follow-up data (*n* = 47, yellow and blue), and participants with both a viable term age scan and follow-up data (*n* = 40, yellow)

A total of 293 V/EPT infants were recruited between 2016 and 2022 for ongoing prospective cohort studies at Children’s National Hospital on the impact of preterm birth on the developing brain and subsequent neurodevelopment. Infants were excluded if they lacked a TEA scan (*n* = 17), the fMRI scan had excessive motion artifacts (*n* = 81), or the infant rated above “mild brain injury” on the Kidokoro scale [47] as determined by an experienced pediatric neuroradiologist (J.M.) (*n* = 47). Follow-up data at 18 months corrected age was only available for a subsample of the total cohort. The final sample consisted of 148 participants possessing both acceptable fMRI scan and procedure data with an average of 6:40 ± 1:45 min of viable resting state data. Importantly, while those not meeting inclusion criteria were slightly younger than included infants (26.85 vs 27.94 weeks, *t*(291) = 3.178, *p* = 0.002), there was no difference in the total number of skin breaks prior to term age (98 vs 112, *p* = 0.208). Based on the initial sample, multiple subsamples were utilized for follow-up analyses. Namely, a subset of possessing both procedures and follow-up data (*n* = 47) and a second subset possessing MRI scan and follow-up data (*n* = 41) (Fig. 1). Finally, an additional 99 term-born infants were recruited as controls (Table 1). The study was approved by the Children’s National Hospital Institutional Review Board (IRB) and performed within regulations and guidelines of the IRB.

**Table 1:** Demographics and clinical characteristics

	Term (*n* = 99)	Preterm (*n* = 148)	*P* value
Gestational at birth (weeks)	39.2 (1.3)	28.02 (2.8)	< .001
Postmenstrual age at scan (weeks)	40.9 (1.3)	40.1 (2.0)	< .001
Age at scan (weeks)	1.6 (0.9)	12.0 (3.3)	< .001
Sex (% male)	55.6	45.4	.117
Maternal characteristics			
Maternal age (years)	34.1 (5.8)	28.0 (5.6)	< .001
Gravida	2 [1, 9]	2 [1, 8]	.058
Primipara (% yes)	55.6	77.2	.001
Education level (%)			< .001
< HS	6.6	9.8	
HS	12.1	32.6	
Some college	19.8	27.2	
College degree	18.7	19.6	
Graduate degree	42.9	10.9	
Employment (%)			.087
Professional	61.5	41.3	
Skilled/clerical/sales	13.2	18.5	
Unemployed/homemaker	25.3	40.2	
Race and Ethnicity (%)			< .001
American/Alaskan Indian	2.0	1.3	
Asian/Pacific Islander	4.0	16.5	
Non-Hispanic Black	39.4	27.8	
Hispanic	11.1	1.3	
Non-Hispanic White	40.4	53.2	
Other	3.0	0	
APGAR at 1 min	8 [6, 9]	4 [0,9]	< .001
APGAR at 5 min	9 [8, 10]	7 [0,9]	< .001
NICU care clinical care			
Number of NICU procedures	-	111 (93.2)	
Length of NICU stay (days)	-	67.7 (34.9)	
Opioid pain medication in NICU (%)	-	79.7	
Fentanyl exposure (%)	-	54.0	
Morphine exposure (%)	-	41.6	
Benzodiazepine medication in NICU (%)	-	27.9	
Dexmedetomidine in NICU (%)	-	15.0	
Underwent surgery prior to TEA (%)	-	30.7	
Ventilated in NICU (%)	-	12.1	
Rates of necrotizing enterocolitis (%)	-	11.4	
Rates of patent ductus arteriosus (%)	-	29.2	
Rates of bronchopulmonary dysplasia (%)	-	21.4	

Numerical data presented as mean (sd) or median [range]

Reported *p* values are from independent *t*-tests for continuous variables and *χ*^2^ for categorical

*HS* High school,* NICU* Neonatal intensive care unit

### Measures and behavioral assessments

A retrospective chart review was performed to catalogue painful procedures experienced during their NICU stay. Consistent with prior literature, we utilized the total number of skin break procedures which included the number of heel sticks, venipunctures, and IV placements [48–50].

Caregivers and infants returned at approximately 18 months postnatal for developmental testing. They completed the Modified Checklist for Autism in Toddlers-Revised (MCHAT), a 20-item assessment utilized as an early screen for autism [51]. The MCHAT score was categorized into levels of autism risk: “not at risk” (< 3) and “at risk” (≥ 3). Caregivers also complete the Mullen Scales of Early Learning (MSEL). The MSEL assesses neurodevelopment from infancy through toddlerhood with scales for gross and fine motor, visual, and receptive and expressive language development [52] and is significantly associated of ASD risk at 18–36 months [53]. While the MCHAT is a clinical screener resulting in difficulty capturing variations in typically developing children who score 0–1, the MSEL is normed using the general population and thus captures typical variability in neurodevelopment.

### MRI acquisition and preprocessing

All MRI images were acquired using a 3 T GE scanner (Discovery MR750, GE Healthcare, Milwaukee, WI) using an 8-channel infant head coil as part of the research protocol. To minimize motion, infants were fed, swaddled in a warm blanket, secured using an infant vacuum pillow, and provided with ear protection consisting of silicone ear plugs and adhesive earmuffs. Infants were not otherwise sedated. All infants were unventilated at time of TEA scan. Anatomical T2 weighted 3D fast-spin echo structural images were collected with the following parameters: 2502 ms TR, 84.37 ms TE, 0.5 × 1.0 × 0.5 mm voxel size, 90° flip angle. Resting state scans were collected with 2000 ms TR, 25 ms TE, 2.5 × 2.5 × 3.2 mm voxel size, 60° flip angle, 100 cm field of view, anterior-to-posterior phase encoding, and 10 min scan duration (300 volumes).

Resting state data were preprocessed using a modified previously published pipelines [54] using Analysis of Functional NeuroImages (AFNI) [55]. In brief, scans underwent within-volume motion correction, slice-time correction, dropping the first four volumes. Following, images were despiked, bias-field corrected, and outliers were censored. Functional images were then aligned to their anatomical and normalized to a PMA 40 template [56]. Signal was intensity scaled to a global mode of 1000 and smoothed using a 5 mm full-width half-maximum blur. Demeaned motion was calculated and volumes exceeding 0.2 mm were excluded. Motion, CSF, and white matter signal were regressed out, and the image underwent 0.009–0.08 bandpass filtering. Throughout the processes, visual inspections of the data were performed for noticeable distortions and drop-off including the raw resting state data, following processing, and again to the parcellation template. After preprocessing, only images with at least 4 min of available data were included in the analyses. Prior work has suggested that 4 min of rest is sufficient to elicit functional proto-networks [25, 32] and been used to successfully track changes of functional connectivity from infancy into early childhood [57].

### Experimental design and statistical analysis

Infants were recruited from Children’s National for a prospective cohort study which included MRI scans during the preterm period and at TEA. Infants were then followed longitudinally to evaluate neurodevelopment, which included a battery of caregiver reports and questionnaires at 18 months of age. Utilizing this cohort, based on infants possessing a high-quality TEA scan as described above, a retrospective chart review was performed to summarize the number of skin break procedures experienced prior to TEA.

**Fig. 2:**
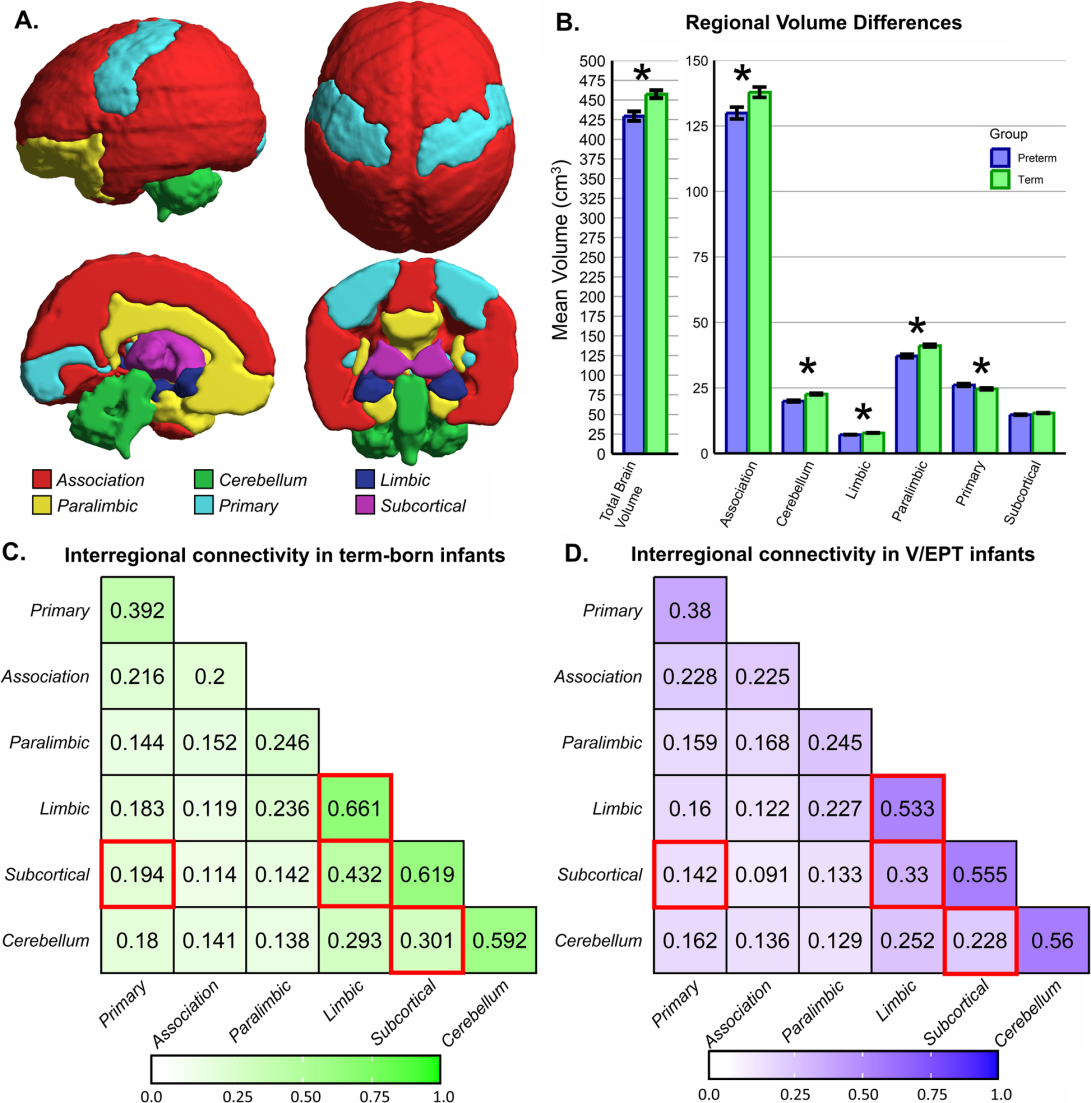
Interregional connectivity differences by birth. **A** Regional parcellation into red-association, green-cerebellum, dark blue-limbic, yellow-paralimbic, light blue-primary, and purple-subcortical. **B** Differences in volume for all regions with all differences significant at *p* < .05. Term-born infants exhibit greater volume in all regions except primary sensorimotor regions which are larger in very and extremely preterm (V/EPT) infants. **C**,**D** Differences in connectivity for all regional connections, with term-born infants exhibiting greater connectivity for four connection highlighted in red: cerebellum-subcortical, limbic-limbic, subcortical-limbic, and subcortical-primary

The mean BOLD time series were extracted from the neonatal aal atlas [56] with the inclusion of additional ROIs comprising the brainstem and cerebellum. Importantly, despite evidence of some functional distinctions across cerebellar lobules [57], given the size of infant cerebellums and our smoothing parameters, we have elected to collapse lobules into cerebellar lobes to minimize signal bleed across ROIS, resulting in a total of 96 ROIs (Additional file 1: Supplemental Information (SI) 1). Pearson correlations were computed between all ROIs and *z*-transformed using a Fischer transformation (Additional file 1: SI 2) [25, 58, 59]. ROIs were grouped into functional regions defined by Mesulam (2000) into primary, association, paralimbic, limbic, subcortical, and cerebellum [60] (Fig. 2A; Additional file 1: SI 3). For structural analyses, the six functional regions were warped into subject space with tissue masking to only include gray matter for cortical ROI. Total parenchymal volume was calculated for the entire brain, followed by volume for each functional region.

First, case–control analyses were performed between the term-born and preterm infants to assess differences in brain volume and connectivity. Group difference ANCOVAs for regional volumes were performed including total brain volume as a covariate to correct for differences across individuals and postmenstrual age to account for the small group difference. The results were then subjected to multiple comparisons correction via false discovery rate (FDR) [61]. Mean interregional connectivity was then compared using multiple independent *t*-tests.

Second, to assess the effect of painful procedures on developing connections, connectivity was correlated with number of skin breaks in V/EPT infants, including intraregional connectivity by assessing connectivity between all ROIs within a region. Then, individual ROI-to-ROI connections exhibiting a significant relationship were identified.

Third, analyses were performed with assessments collected at 18 months of age. Initially, *t*-tests were performed to evaluate differences in skin breaks between ASD risk groups. Due to a purported relationship between autism and prematurity [10, 62] and to assess a dose–response relationship, a regression analysis was then performed examining painful procedures, GA at birth, and the interaction of the two on autism risk, using the MCHAT total score. Following significant interactions, marginal effects for breaks at each week of birth GA were calculated. Next, MSEL subscales were examined in the same manner to assess variation in neurodevelopment.

Finally, analyses were conducted to assess the relationship between connectivity and neurodevelopmental scores. Connections sensitive to breaks at TEA were correlated with neurodevelopmental scores sensitive to breaks at 18 months to assess connectivity-neurodevelopmental overlaps.

## Results

### Study population

The study population includes 247 subjects: 148 infants born V/EPT with mean birth GA of 28.02 ± 2.80 weeks and scanned at 40.10 ± 2.01 weeks PMA and 99 healthy term-born infants from uncomplicated pregnancies with a mean birth GA of 39.15 ± 1.25 weeks and scanned at 40.90 ± 1.31 weeks PMA (Table 1).

### Differences in term and V/EPT infant brain volume and connectivity

Compared to term-born infants, V/EPT infants exhibit significant differences in brain volume and connectivity. Term-born infants display significantly larger brain volumes overall (457.30cm^3^ vs 430.62cm^3^, *t*(238) = 3.485, *p* < 0.001), including larger association, cerebellar, limbic, and paralimbic volumes (*p*_corr_ < 0.001). Convesely, preterm infants exhibit larger primary sensorimotor regions (*p*_corr_ < 0.001) (Fig. 2B, Table 2). Term-born infants exhibit differences in connectivity relative to V/EPT infants, with greater cerebellum-subcortical, limbic-limbic, subcortical-limbic, and subcortical-primary connectivity (*p*_corr_ < 0.037) compared to V/EPT infants (Fig. 2C,D; Additional file 1: SI 4).

**Table 2:** Volumetric group differences

Region	Term	Preterm	Group F	Group *p*	Group *p*_corr_	TBV F	TBV *p*	TBV *p*_corr_	Age F	Age *p*	Age *p*_corr_
Association	137,851.8	129,913.5	138.2	< .001	< .001	4669.1	< .001	< .001	70.17	< .001	< .001
Cerebellum	22,626.67	19,954.29	108.5	< .001	< .001	529.8	< .001	< .001	17.62	< .001	< .001
Limbic	7809.824	7134.252	79.2	< .001	< .001	409.5	< .001	< .001	0.2	.656	.727
Paralimbic	41,120.24	37,188.5	129.9	< .001	< .001	1419.3	< .001	< .001	6.6	.011	.162
Primary	24,625.42	26,126.44	30.0	< .001	< .001	1267.8	< .001	< .001	7.5	.007	< .001
Subcortical	15,394.36	14,775.56	19.9	< .001	< .001	825.0	< .001	< .001	.1	.727	.727

Volumes presented in mm^3^, with corrected *p* values based on FDR correction

### Skin break procedures and brain measures in V/EPT infants

**Fig. 3:**
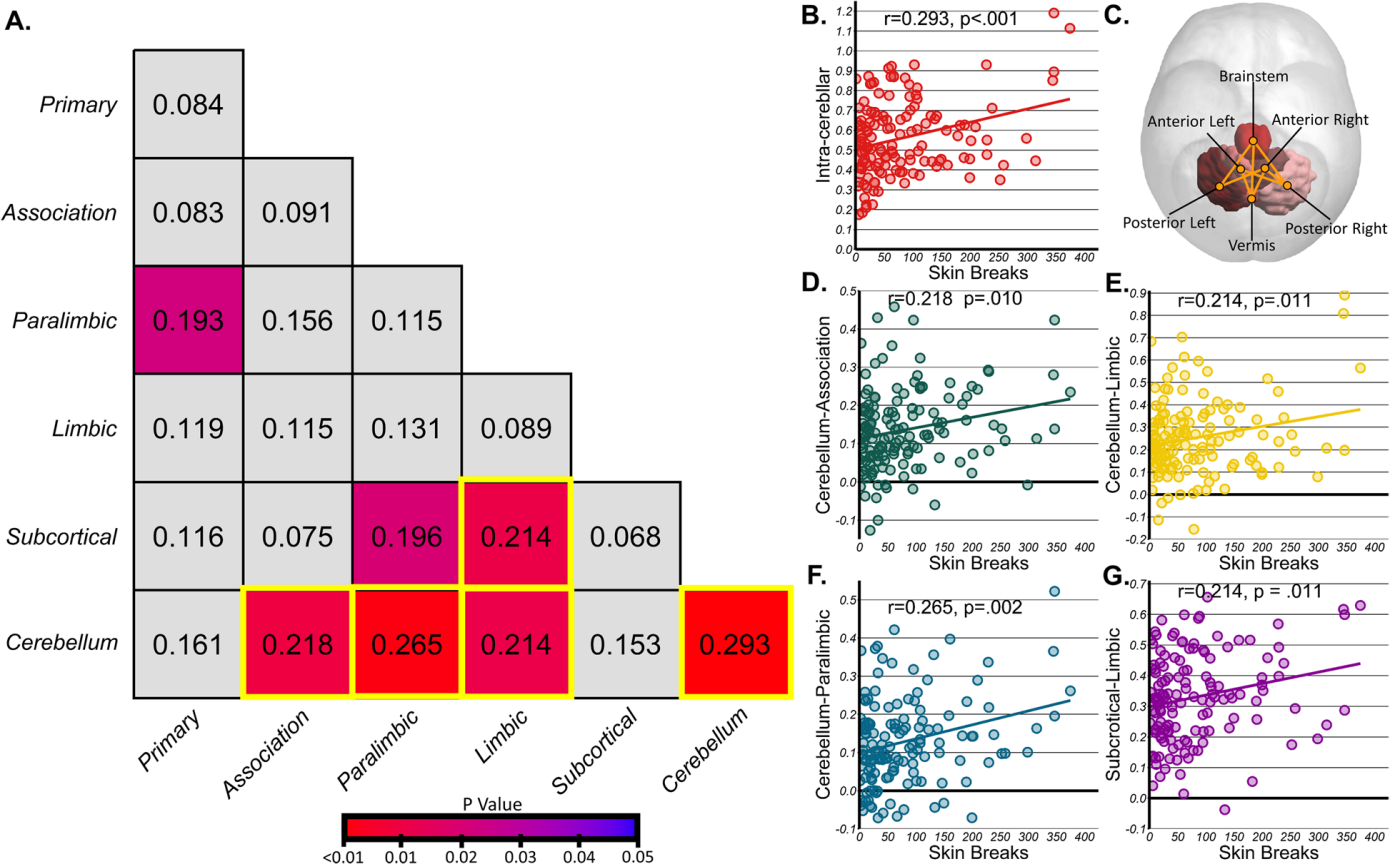
Interregional correlations for connectivity and painful procedures. **A** Interregional correlation *r*-values for each comparison, colored by *p*-value. Following false discovery rate (FDR) correction, cerebellum to association, paralimbic, limbic, and cerebellum as well as subcortical to limbic remained significant and are highlighted in yellow. **B**,**C** Significant relationship for inter-cerebellar connectivity with skin breaks, alongside ROI-to-ROI connections sensitive to pain. **D–G** Significant relationship for other regional connections and breaks. No significant ROI-to-ROI connections survived multiple comparison corrections across these regions

First, no significant relationships were observed between painful procedures and regional volume (*p*_corr_ ≥ 0.341); however, significant relationships were observed with functional connectivity. Following multiple comparison correction, intra-cerebellar connectivity (*r*(138) = 0.293, *p* < 0.001, *p*_corr_ = 0.009), as well as cerebellum connectivity with association (*r*(138) = 0.218, *p* = 0.002, *p*_corr_ = 0.047), paralimbic (*r*(138) = 0.265, *p* < 0.001, *p*_corr_ = 0.016) and limbic (*r*(138) = 0.214, *p* = 0.001, *p*_corr_ = 0.047) regions and subcortical-limbic connectivity (*r*(138) = 0.214, *p* = 0.011, *p*_corr_ = 0.047) exhibited a clear, positive relationship with the number of skin breaks (Fig. 3A). Subsequent control analyses for sociodemographic variables found no relationship between connectivity at term age between regions and maternal race (ps > 0.314), education (ps > 0.150), or employment status (ps > 0.125).

Subsequently, individual ROI-to-ROI comparisons revealed individual connections sensitive to skin breaks. Following correction, 8 intra-cerebellar connections exhibited significant positive relationships (*p*_corr_ < 0.024) with skin breaks (range = 0.210–0.287) (Table 3 and Fig. 3, see Additional file 1: SI 5–9 for the full tables). For the interregional connections, 44 of 264 cerebellum-association, 38 of 144 cerebellum-paralimbic connections, 9 of 36 cerebellum-limbic connections, and 10 of 48 subcortical-limbic connections were exhibited significant positive relationships between breaks and connectivity but none survived multiple comparison correction.

**Table 3:** Significant ROI-to-ROI connections correlated with skin breaks by region

Regions	ROI 1	ROI2	r	*p*	*p*_corr_
Intra-cerebellar	Anterior left	Posterior right	0.323	< .001	.001*
	Posterior left	Brainstem	0.319	< .001	.001*
	Posterior right	Brainstem	0.319	< .001	.001*
	Anterior right	Posterior right	0.297	< .001	.002*
	Anterior left	Vermis	0.250	.003	.010*
	Vermis	Brainstem	0.221	.010	.022*
	Anterior left	Anterior right	0.220	.010	.022*
	Anterior right	Vermis	0.208	.016	.029*
Cerebellum-limbic	Posterior left	Rectus left	0.330	< .001	.013*
	Vermis	Parahippocampal gyrus right	0.300	< .001	.027*
	Posterior left	Rectus right	0.292	< .001	.027*
	Posterior left	Orbital (superior) left	0.281	< .001	.027*
	Brainstem	Anterior cingulate left	0.275	.001	.027*
	Posterior left	Orbital (superior) right	0.275	.001	.027*
	Posterior right	Parahippocampal gyrus right	0.273	.001	.027*
Cerebellum-paralimbic	Posterior right	Olfactory right	0.267	.002	.036*
	Vermis	Olfactory right	0.257	.003	.036*
	Posterior left	Olfactory left	0.445	.004	.036*
	Anterior right	Olfactory right	0.241	.004	.036*
	Posterior left	Amygdala left	0.241	.005	.036*
	Vermis	Olfactory left	0.226	.009	.044*
	Posterior left	Olfactory right	0.225	.009	.044*

^*^ Significant at *p* < .05 corrected

### Skin break procedures and neurodevelopmental outcomes

**Fig. 4:**
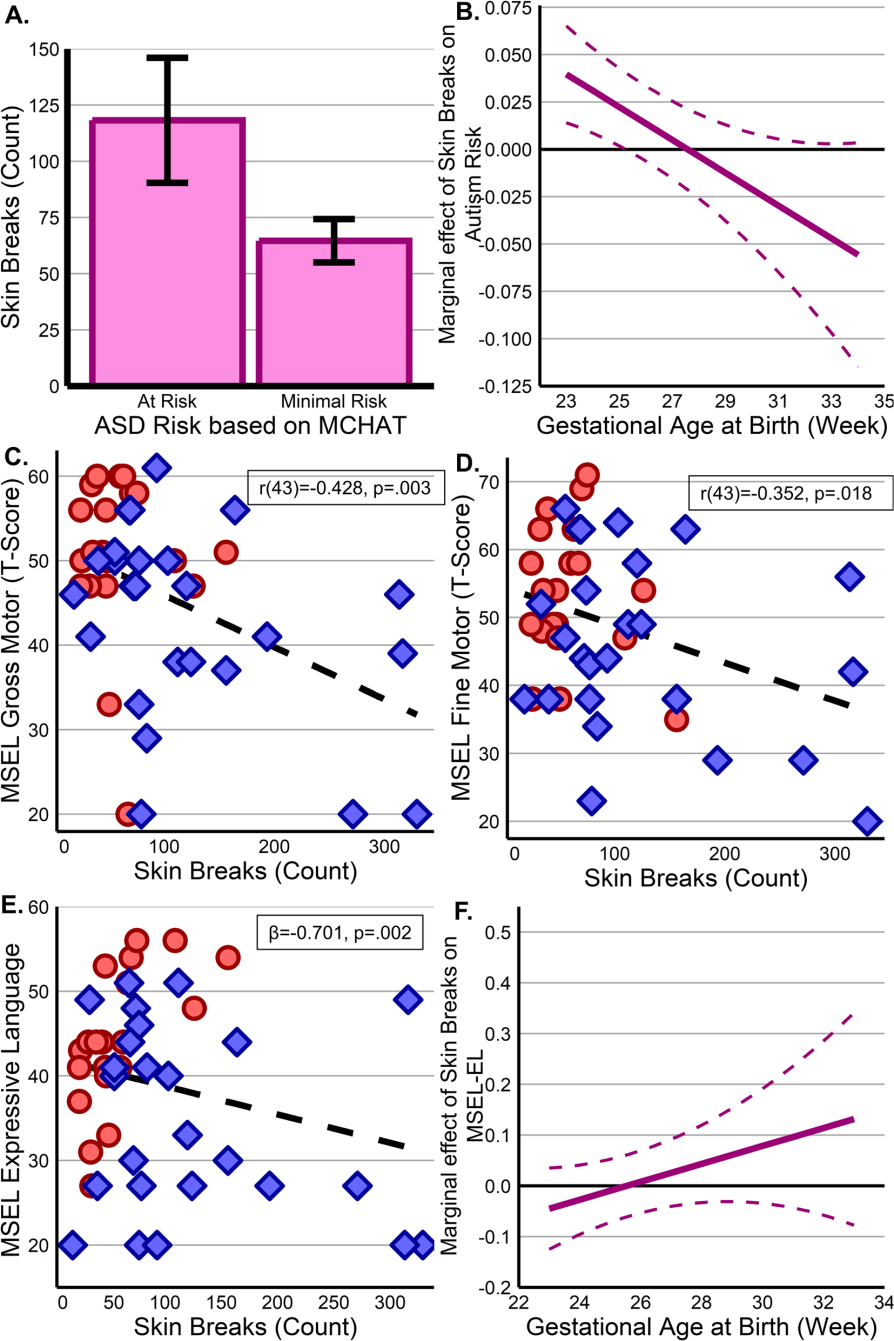
Impact of skin break procedures on neurodevelopment. **A** Infants at risk for autism spectrum disorder (ASD) experienced significantly more procedures than those not as risk with an apparently age effect (**B**) where earlier birth GA is positive associated between skin breaks and ASD risk score. **C,D** MSEL Gross and Fine motor scales exhibit a clear relationship of increasing deficits with greater pain exposures, showing EPT infants with blue diamonds and VPT infants with red circles. **E** A similar effect is observable in the expressive language scale which also shows a prominent age effect (**F**) with earlier birth GA showing a relationship between painful procedures and greater impairment (lower scores) which lessens over time

At 18 months of age, toddlers identified as at risk for autism based on the MCHAT experienced significantly more breaks (mean = 118.27 ± 92.26), than those not at risk (mean = 64.65 ± 56.31, *t*(43) = 2.327, *p* = 0.024) (Fig. 4A). A subsequent regression model was significant (*R*^2^_adj_^=^0.329, *F*(3,41) = 6.701, *p* < 0.001). The model found that there is both a significant main effect for skin breaks (*β* = 0.175, *p* = 0.023) and an interaction between skin breaks and GA at birth (*β* =  − 0.006, *p* = 0.035), but GA alone was not significant (*p* = 0.833). Evaluating marginal effects of skin breaks at each week of birth GA, skin breaks are positively associated with MCHAT score at younger birth ages, suggesting the most prematurely born infants are more vulnerable (Fig. 4B). Subsequent follow-up analyses controlling for sociodemographic variables found these results were present when controlling for the impact of maternal race, education, and employment status (Additional file 1: SI 10).

Consistent with prior literature [53], all MSEL subscales were strongly correlated with the MCHAT (range =  − 0.548 to − 0.677, ps < 0.001) with higher MCHAT scores associated with greater impairment on MSEL subscales. While there were no relationships between breaks and visual reception or receptive language (ps > 0.261), simple relationships were observed with gross (*r*(43) =  − 0.428, *p* = 0.003) and fine (*r*(43) =  − 0.352, *p* = 0.018) motor scales (Fig. 4C,D), with no birth GA or interaction effects observable. Alternatively, expressive language exhibited the same complex relationship as the MCHAT (*R*^2^_adj_^=^0.402, F(3,41) = 9.191, *p* < 0.001), with a main effect for exposures (*β* =  − 0.701, *p* = 0.002) (Fig. 4E) and interaction (*β* = 0.027, *p* = 0.001), but no effect for GA alone (*p* = 0.471). Marginal effects suggest that pain exposure early in life is negatively associated with expressive language—an effect that is minimized with increasing age (Fig. 4F). Follow-up analyses found these relationships to be durable even when accounting for maternal race, education, and employment status (Additional file 1: SI 9).

### Connectivity and longitudinal neurodevelopment

Finally, analyses were performed to assess associations between TEA brain connectivity and 18-month neurodevelopment. Both MCHAT and Mullens scores were then compared to the 8 cerebellar ROI-to-ROI pairs associated with skin breaks. MCHAT scores were not associated with term age connectivity of any of the cerebellar connections (ps > 0.248). Similarly, no relationship was observed between cerebellar connectivity and either gross motor (ps > 0.228) or fine motor (ps > 0.528) scores at 18 months. Conversely, expressive language was associated with connectivity strength at term equivalent age between the anterior left and posterior left (*r*(38) =  − 0.388, *p* = 0.016, *p*_corr_ = 0.048) anterior left and vermis (*r*(38) =  − 0.447, *p* = 0.005, *p*_corr_ = 0.040), and anterior right and vermis (*r*(38) =  − 0.381, *p* = 0.018, *p*_corr_ = 0.48) (Table 4). Negative relationships for each connection suggest that greater connectivity is associated with lower expressive language skills at 18 months.

**Table 4:** Associations between connectivity and 18-month follow-up

	Cerebellar ROI 1	Cerebellar ROI 2	*r*	*p*	*p*_corr_
MCHAT	Anterior left	Posterior left	0.173	.313	.453
	Anterior left	Vermis	0.146	.396	.453
	Anterior left	Posterior right	0.197	.248	.453
	Anterior right	Posterior left	0.018	.919	.919
	Anterior right	Vermis	0.158	.356	.453
	Posterior left	Brainstem	0.173	.313	.453
	Vermis	Brainstem	0.146	.396	.453
	Posterior right	Brainstem	0.197	.248	.453
MSEL Gross Motor	Anterior left	Posterior left	0.035	.836	.836
	Anterior left	Vermis	− 0.071	.676	.836
	Anterior left	Posterior right	0.105	.538	.836
	Anterior right	Posterior left	0.042	.805	.836
	Anterior right	Vermis	− 0.203	.228	.836
	Posterior left	Brainstem	0.057	.738	.836
	Vermis	Brainstem	− 0.085	.618	.836
	Posterior right	Brainstem	0.047	.781	.836
MSEL Fine Motor	Anterior left	Posterior left	− 0.071	.677	.864
	Anterior left	Vermis	0.033	.847	.864
	Anterior left	Posterior right	0.107	.528	.864
	Anterior right	Posterior left	0.067	.695	.864
	Anterior right	Vermis	− 0.029	.864	.864
	Posterior left	Brainstem	− 0.043	.801	.864
	Vermis	Brainstem	− 0.083	.623	.864
	Posterior right	Brainstem	0.044	.798	.864
MSEL Expressive Language	Anterior left	Posterior left	− 0.388	.016	.048*
	Anterior left	Vermis	− 0.447	.005	.040*
	Anterior left	Posterior right	− 0.247	.135	.221
	Anterior right	Posterior left	− 0.245	.138	.221
	Anterior right	Vermis	− 0.381	.018	.048*
	Posterior left	Brainstem	− 0.074	.659	.720
	Vermis	Brainstem	− 0.214	.197	.263
	Posterior right	Brainstem	− 0.060	.720	.720

Correlation table for the Modified Checklist for Autism in Toddlers (MCHAT) and Mullens Scales of Early Learning (MSEL) scores at 18 months with ROI-to-ROI connectivity sensitive to pain at term equivalent age. ^*^Significant at *p* < .05 corrected

## Discussion

Our findings provide four major advances in the understanding of early-life painful exposures in preterm infants. First, exposures are associated with alterations in functional connectivity as early as TEA. Second, exposures are associated with worse neurodevelopmental outcomes including motor and language deficits and autism risk. Third, there may be an early critical period of vulnerability to pain. And fourth, connectivity within pain-sensitive connections may be predictive of future neurodevelopmental impairments.

Our cerebellar results harmonize with previous research showing atypical development in V/EPT infants, with smaller cerebellar volumes at TEA [22, 28–30]. This may be due to relative early cerebellar development, which experiences rapid growth in the second trimester [63] and can quadruple in volume prior to birth [30]. Between rapid structural growth and acting as an early functional hub [58], the cerebellum is a logical focus for understanding early disruptions in brain development.

The differences in cerebellum connectivity may stem from its role in the brain’s pain matrix. The cerebellum, together with the primary somatosensory cortex, limbic and paralimbic regions, including the amygdala, ventral striatum, and insula [64–67] make up the backbone of the mature, adult pain system [68]. While this network is fully intact in childhood, most functional networks are just beginning to organize during late gestation [26, 69–71]. While not directly causative, we may be observing early disruptions in this network’s formation with co-activations through repeated painful exposures. This interpretation would strongly comport with findings in adults, where chronic pain is associated with hyper-connectivity between pain regions, including the cerebellum, brainstem, and prefrontal regions [72, 73] (for review see Mayer et al. [74]). Our findings mirror chronic pain in adults, where repeated experiences of pain are associated with increases in connectivity. It should be noted, however, that other reports emphasize changes in the medial and inferior frontal lobes in both humans [72, 73] and rodents [46]. Conversely, our results largely implicate only the inferior frontal lobe. While not clear, this may be due to the relative underdevelopment of the region at later gestational ages (GA), making these more immature connections easier to influence than more established regions. Although more work is required to directly assess causal mechanisms between painful experiences and alterations in connectivity, greater connectivity is believed to be a proxy for the degree of synchronicity between two regions, derived from consistent and repeated coactivation. Therefore, functional connections are generally thought to form and strengthen in an experience-driven manner [75]. Importantly, the observed differences are not simply brain-wide increases in connectivity, but instead appear to be heavily localized within pain regions, further suggesting the changes are likely associated the repeated coactivation of the pain matrix when exposed to painful experiences. Importantly, this pattern extends beyond the broad, functional dysmaturation often observed in premature infants compared to term born both prior to TEA [22, 76] as well as at and beyond TEA [77], and instead suggest that pain exposures are associated with greater connectivity between selective regions that far outpaces the deficits seen between term and preterm born infants across other regions.

In our study cerebellar alterations at TEA were associated with development at 18 months. Altered cerebellar volume and connectivity are a consistently reported feature in ASD [78–80], including hyperconnectivity between the cerebellum and inferior frontal regions [81–83]. In addition, disrupted limbic connectivity has been associated with deficits in ASD including social and executive function [84, 85]. Therefore, pronounced alterations in the connections of these regions may be mimicking autistic-like differences. Additionally, our findings in expressive language are bolstered by growing evidence in the importance of the cerebellum in language, especially language production [86] and has similarly been linked to ASD [87]. While this cohort will continue to be assessed throughout toddlerhood and early childhood, these findings represent important associations to be followed as more infants reach assessment ages to validate the significance of these findings in the larger cohort.

Finally, there may be a particularly vulnerable period for EPT infants during which pain exposure may elevate the risk of neurodevelopmental deficits. Our findings corroborate prior work on the worsening impacts of prematurity on structural brain development [88]. The most likely reason being the lagging development of pain modulation pathways relative to afferent nociceptive pathways. Additionally, due to the rostral to caudal development of the brain in third trimester [89, 90], the most rostral frontal regions may be the most vulnerable to insult as they are still more actively developing than more posterior regions.

Despite the strengths of this study, there are some limitations. First, there is no standard way to measure NICU pain, with highly variable ratings across raters and between subjective, physiological, and behavioral measures [91–93]. Skin breaks serve as a common procedure that is widely accepted to cause pain/discomfort that occur frequently as part of routine care and have been linked with development of pain pathways in both human and animal studies [49, 88, 94]. Given the known discomfort associated with skin breaks, routine care often employees nonpharmacological interventions such as kangaroo care [95] swaddling or pacifier use to temper discomfort [96, 97]. Such interventions are difficult to quantify and are unaccounted for in this work. Furthermore, pharmacologic interventions, such as morphine, are also known to adversely influence brain development, and as such seldom employed as routine interventions prior to common skin break procedures. Similarly, we utilized caregiver report and questionnaires at 18 months rather than clinician-driven neurodevelopmental assessments. Future prospective studies should collect and control for both forms of pain management [95] as well as utilize clinical neurodevelopmental assessments. Second, in some cases multiple skin breaks may be required to successfully complete a single procedure, such as requiring a second heel stick to successfully draw blood. Multiple attempts for skin break procedures are variably recorded (routine for central line and intravenous placement, but not for heel lance) and, as such, could not be fully accounted for in our analyses. Third, the neurodevelopmental outcomes reported likely reflect both the effects of skin break procedures, as well as illness severity. Infants with higher disease burden require more consistent monitoring including more heel sticks and receive more interventions. This significant collinearity cannot be adequately controlled in the presented study design due to the tight coupling between the variables. As a result, we are unable to fully disentangle the impact of disease burden and the frequent experience of invasive skin break procedures. However, there is evidence that painful experiences are associated with alterations in brain connectivity [72, 73]. As a result, it is likely a contributor to altered neurodevelopmental trajectories that, unlike disease severity, is a more easily modifiable experience that can be targeted by interventions to optimally support neurodevelopment even in cases of significant illness. Fourth, pain is only one of several sensory experiences that are heightened in the extrauterine versus intrauterine environment. While pain is an intuitive mechanism driving these changes, extreme auditory and visual stimuli likely also impact development but were not accounted for here. Future studies should explore their impact on development. Finally, our subsample analyses linking connectivity to neurodevelopment are likely underpowered to detect more subtle relationships, especially as many of our observed correlations are between 0.2 and 0.4. Brain-behavior connections are often complex even when not assessed with 18-month lag and report correlation values within the observed range [98–100]. More work is required, however, to properly assess the longitudinal effects of painful exposures and interventions at all stages of development. Finally, social and societal risk factors are thought to play a significant role in neurodevelopment. While our findings remain durable when controlling for a subset of sociodemographic, a more robust collection of a wide range of socioeconomic data should be collected in the future to assess its interaction with pain and long-term development.

## Conclusions

These findings provide an important step in understanding the impacts of earlier-than-typical exposure to the extrauterine environment. Our results suggest that early, repeated exposures to pain have a significant impact on the developing functional connectome and neurodevelopmental outcomes. These observed differences have important implications for our wider understanding of the effect of painful NICU procedures, which are often performed in the absence of analgesia, and how we conceptualize the long-term risks of these procedures. Finally, these findings emphasize the need to anticipate and develop novel strategies to treat pain given the known adverse effects of traditional, opioid-based pharmacotherapies.

### Supplementary Information

**Additional file 1: Supplemental Information 1. **Full Brain Parcellation.  **Supplemental Information 2. **Z-score Transformation. **Supplemental Information 3.** ROI and regional classifications. **Supplemental Information 4.** All Regional Connections.

## Data Availability

The datasets used and/or analyzed during the current study are available from the corresponding author on reasonable request.

## Abbreviations

V/EPT: Very and extremely premature
VPT: Very preterm
EPT: Extremely preterm
PMA: Postmenstrual age
ASD: Autism spectrum disorder
NICU: Neonatal intensive care units
TEA: Term-equivalent age
MCHAT: Modified checklist for autism in toddlers-revised
MSEL: Mullen scales of early learning
AFNI: Analysis of functional neuroImages
FDR: False discovery rate
GA: Gestational age
