# Supplementary material for: Experience of early-life pain in premature infants is associated with atypical cerebellar development and later neurodevelopmental deficits
Source: BMC Med. 2023 Nov 14;21:435. doi: 10.1186/s12916-023-03141-w (PMC10644599; doi:10.1186/s12916-023-03141-w)
Supplement: Supplementary file 1 — Additional file 1: Supplemental Information 1. Full Brain Parcellation. Supplemental Information 2. Z-score Transformation. Supplemental Information 3. ROI and regional classifications. Supplemental Information 4. All Regional Connections. [file 12916_2023_3141_MOESM1_ESM.docx]

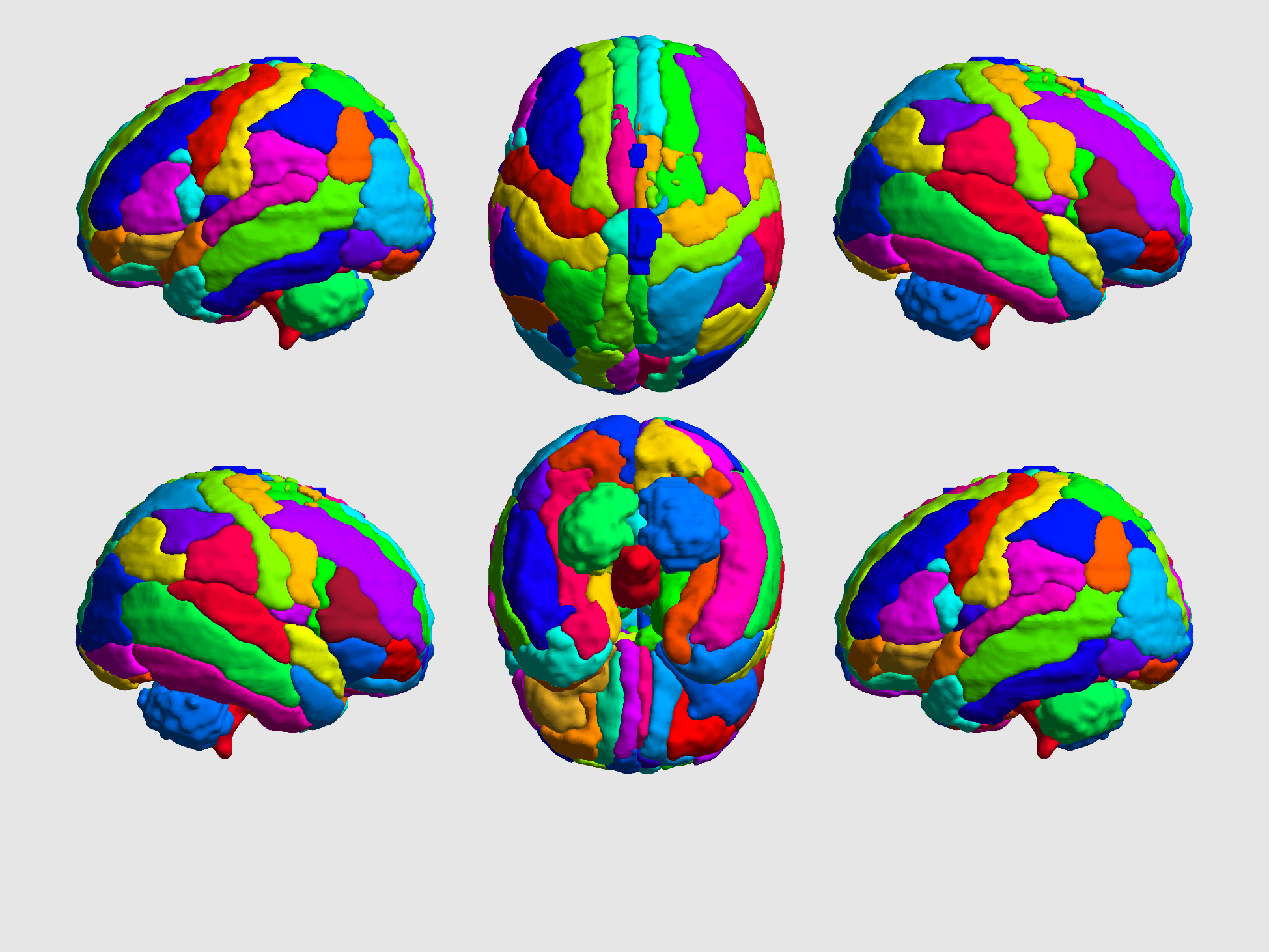


**Supplemental Information 1: Full Brain Parcellation**

*Full 96 ROI atlas left, right, mid sagittal, and coronal sections.*

**
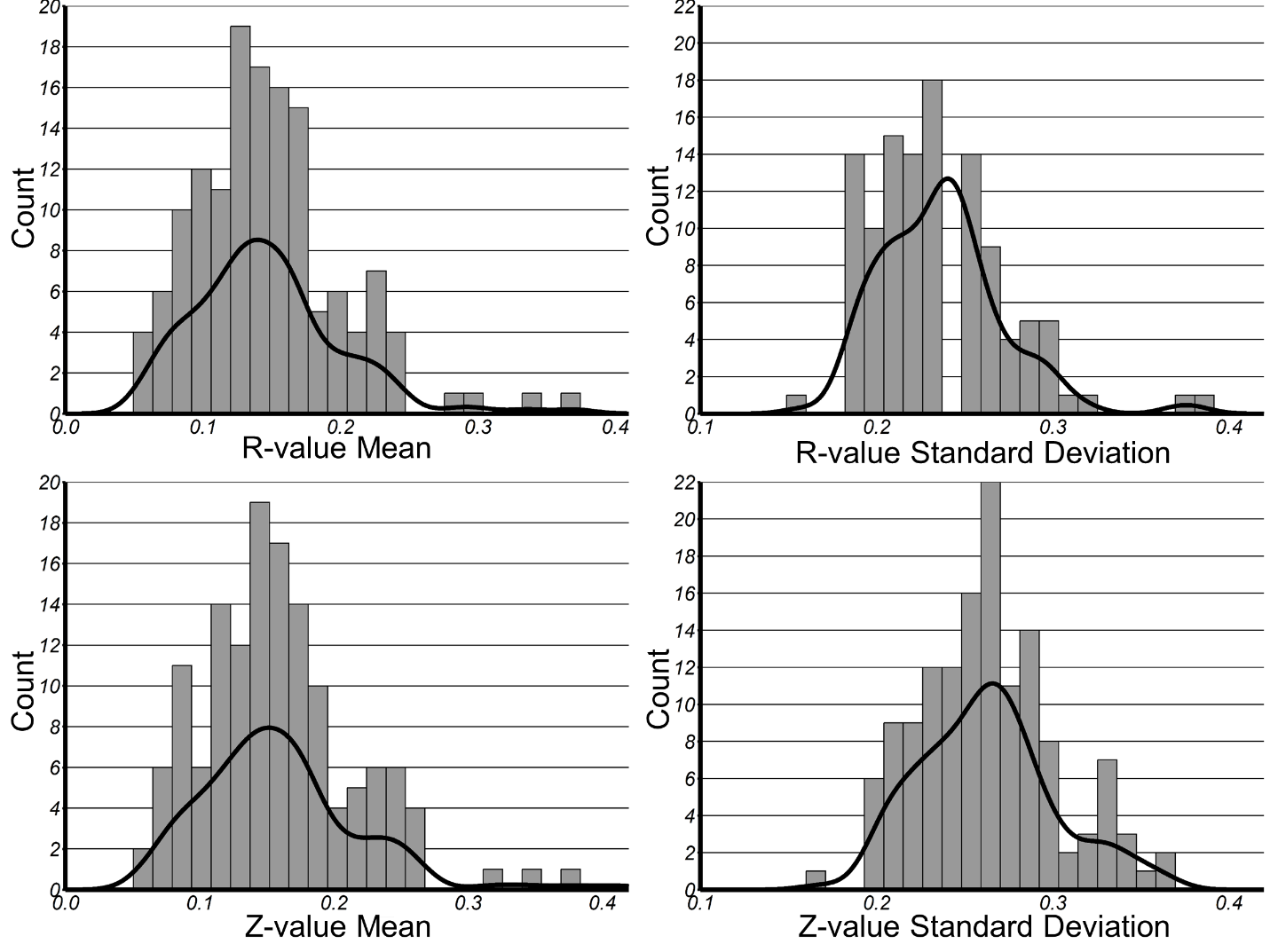
**

**Supplemental Information 2: Z-score Transformation**

Distributions of mean connectivity and standard deviation before and after Fischer-z transformations. Prior to transformations, participants exhibited a mean connectivity of 0.1467 with a standard deviation of 0.2364. Following transformation, we observed a mean of 0.1599 and standard deviation of 0.2652.

| Supplemental Information 3: ROI and regional classifications | | |
| --- | --- | --- |
| ROI | Label | Functional Region |
| 1 | Precentral gyrus Left | Primary |
| 2 | Precentral gyrus Right | Primary |
| 3 | Superior frontal gyrus (dorsal) Left | Association |
| 4 | Superior frontal gyrus (dorsal) Right | Association |
| 5 | Orbitofrontal cortex (superior) Left | Paralimbic |
| 6 | Orbitofrontal cortex (superior) Right | Paralimbic |
| 7 | Middle frontal gyrus Left | Association |
| 8 | Middle frontal gyrus Right | Association |
| 9 | Orbitofrontal cortex (middle) Left | Paralimbic |
| 10 | Orbitofrontal cortex (middle) Right | Paralimbic |
| 11 | Inferior frontal gyrus (opercular) Left | Association |
| 12 | Inferior frontal gyrus (opercular) Right | Association |
| 13 | Inferior frontal gyrus (triangular) Left | Association |
| 14 | Inferior frontal gyrus (triangular) Right | Association |
| 15 | Orbitofrontal cortex (inferior) Left | Paralimbic |
| 16 | Orbitofrontal cortex (inferior) Right | Paralimbic |
| 17 | Rolandic operculum Left | Association |
| 18 | Rolandic operculum Right | Association |
| 19 | Supplementary motor area Left | Association |
| 20 | Supplementary motor area Right | Association |
| 21 | Olfactory Left | Limbic |
| 22 | Olfactory Right | Limbic |
| 23 | Superior frontal gyrus (medial) Left | Association |
| 24 | Superior frontal gyrus (medial) Right | Association |
| 25 | Orbitofrontal cortex (medial) Left | Paralimbic |
| 26 | Orbitofrontal cortex (medial) Right | Paralimbic |
| 27 | Rectus gyrus Left | Paralimbic |
| 28 | Rectus gyrus Right | Paralimbic |
| 29 | Insula Left | Paralimbic |
| 30 | Insula Right | Paralimbic |
| 31 | Anterior cingulate gyrus Left | Paralimbic |
| 32 | Anterior cingulate gyrus Right | Paralimbic |
| 33 | Middle cingulate gyrus Left | Paralimbic |
| 34 | Middle cingulate gyrus Right | Paralimbic |
| 35 | Posterior cingulate gyrus Left | Paralimbic |
| 36 | Posterior cingulate gyrus Right | Paralimbic |
| 37 | Hippocampus Left | Limbic |
| 38 | Hippocampus Right | Limbic |
| 39 | Parahippocampal gyrus Left | Paralimbic |
| 40 | Parahippocampal gyrus Right | Paralimbic |
| 41 | Amygdala Left | Limbic |
| 42 | Amygdala Right | Limbic |
| 43 | Calcarine cortex Left | Primary |
| 44 | Calcarine cortex Right | Primary |
| 45 | Cuneus Left | Association |
| 46 | Cuneus Right | Association |
| 47 | Lingual gyrus Left | Association |
| 48 | Lingual gyrus Right | Association |
| 49 | Superior occipital gyrus Left | Association |
| 50 | Superior occipital gyrus Right | Association |
| 51 | Middle occipital gyrus Left | Association |
| 52 | Middle occipital gyrus Right | Association |
| 53 | Inferior occipital gyrus Left | Association |
| 54 | Inferior occipital gyrus Right | Association |
| 55 | Fusiform gyrus Left | Association |
| 56 | Fusiform gyrus Right | Association |
| 57 | Postcentral gyrus Left | Primary |
| 58 | Postcentral gyrus Right | Primary |
| 59 | Superior parietal gyrus Left | Association |
| 60 | Superior parietal gyrus Right | Association |
| 61 | Inferior parietal lobule Left | Association |
| 62 | Inferior parietal lobule Right | Association |
| 63 | Supramarginal gyrus Left | Association |
| 64 | Supramarginal gyrus Right | Association |
| 65 | Angular gyrus Left | Association |
| 66 | Angular gyrus Right | Association |
| 67 | Precuneus Left | Association |
| 68 | Precuneus Right | Association |
| 69 | Paracentral lobule Left | Association |
| 70 | Paracentral lobule Right | Association |
| 71 | Caudate Left | Subcortical |
| 72 | Caudate Right | Subcortical |
| 73 | Putamen Left | Subcortical |
| 74 | Putamen Right | Subcortical |
| 75 | Pallidum Left | Subcortical |
| 76 | Pallidum Right | Subcortical |
| 77 | Thalamus Left | Subcortical |
| 78 | Thalamus Right | Subcortical |
| 79 | Heschl gyrus Left | Primary |
| 80 | Heschl gyrus Right | Primary |
| 81 | Superior temporal gyrus Left | Association |
| 82 | Superior temporal gyrus Right | Association |
| 83 | Temporal pole (superior) Left | Paralimbic |
| 84 | Temporal pole (superior) Right | Paralimbic |
| 85 | Middle temporal gyrus Left | Association |
| 86 | Middle temporal gyrus Right | Association |
| 87 | Temporal pole (middle) Left | Paralimbic |
| 88 | Temporal pole (middle) Right | Paralimbic |
| 89 | Inferior temporal gyrus Left | Association |
| 90 | Inferior temporal gyrus Right | Association |
| 91 | Anterior Lobe Left | Cerebellar-Brainstem |
| 92 | Anterior Lobe Right | Cerebellar-Brainstem |
| 93 | Posterior Lobe Left | Cerebellar-Brainstem |
| 94 | Posterior Lobe Right | Cerebellar-Brainstem |
| 95 | Vermis | Cerebellar-Brainstem |
| 96 | Brainstem | Cerebellar-Brainstem |

| Supplemental Information 4: All Regional Connections | | | | | | |
| --- | --- | --- | --- | --- | --- | --- |
| Region 1 | Region 2 | Term | Preterm | t | p | p_corr_ |
| Primary | Primary | 0.392 | 0.380 | 0.554 | .580 | .692 |
| Primary | Association | 0.216 | 0.228 | -0.864 | .389 | .583 |
| Primary | Paralimbic | 0.144 | 0.159 | -1.147 | .253 | .498 |
| Primary | Limbic | 0.183 | 0.160 | 1.091 | .277 | .498 |
| Primary | Subcortical | 0.194 | 0.142 | 2.720 | .007 | .037* |
| Primary | Cerebellum | 0.180 | 0.162 | 0.935 | .351 | .574 |
| Association | Association | 0.200 | 0.225 | -2.112 | .036 | .144† |
| Association | Paralimbic | 0.152 | 0.168 | -1.472 | .143 | .343 |
| Association | Limbic | 0.119 | 0.122 | -0.213 | .832 | .856 |
| Association | Subcortical | 0.114 | 0.091 | 1.674 | .096 | .266 |
| Association | Cerebellum | 0.141 | 0.136 | 0.368 | .713 | .778 |
| Paralimbic | Paralimbic | 0.246 | 0.245 | 0.118 | .906 | .906 |
| Paralimbic | Limbic | 0.236 | 0.227 | 0.532 | .596 | .692 |
| Paralimbic | Subcortical | 0.142 | 0.133 | 0.676 | .500 | .692 |
| Paralimbic | Cerebellum | 0.138 | 0.129 | 0.568 | .571 | .692 |
| Limbic | Limbic | 0.661 | 0.533 | 4.779 | < .001 | < .001* |
| Limbic | Subcortical | 0.432 | 0.330 | 4.687 | < .001 | < .001* |
| Limbic | Cerebellum | 0.293 | 0.252 | 1.724 | .086 | .266 |
| Subcortical | Subcortical | 0.619 | 0.555 | 2.507 | .013 | .059† |
| Subcortical | Cerebellum | 0.301 | 0.228 | 3.321 | .001 | .008* |
| Cerebellum | Cerebellum | 0.592 | 0.560 | 1.174 | .242 | .498 |
| Full t test table for preterm/term differences in interregional connectivity. Significant connections are highlighted yellow if they survive FDR correction and blue if they were significant prior to correction but not after | | | | | | |

| Supplemental Information 5: All Intra-Cerebellar-Brainstem ROI Connections | | | | |
| --- | --- | --- | --- | --- |
| ROI 1 | ROI 2 | r | p | p_corr_ |
| Anterior Left | Anterior Right | 0.138 | .105 | .157 |
| Anterior Left | Brainstem | 0.100 | .241 | .278 |
| Anterior Left | Posterior Left | 0.271 | .001 | .005* |
| Anterior Left | Posterior Right | 0.210 | .013 | .024* |
| Anterior Left | Vermis | 0.274 | .001 | .005* |
| Anterior Right | Brainstem | 0.078 | .360 | .386 |
| Anterior Right | Posterior Left | 0.258 | .002 | .006* |
| Anterior Right | Posterior Right | 0.126 | .138 | .172 |
| Anterior Right | Vermis | 0.228 | .007 | .017* |
| Posterior Left | Brainstem | 0.287 | < .001 | .005* |
| Posterior Left | Posterior Right | 0.007 | .935 | .935 |
| Posterior Left | Vermis | 0.128 | .131 | .172 |
| Posterior Right | Brainstem | 0.274 | .001 | .005* |
| Posterior Right | Vermis | 0.143 | .092 | .154† |
| Vermis | Brainstem | 0.219 | .009 | .020* |
| Full correlation table for cerebellar-brainstem and paralimbic ROIs. Significant connections are highlighted yellow if they survive FDR correction and blue if they were significant prior to correction but not after | | | | |

| Supplemental Information 6: All Subcortical and Limbic ROI Connections | | | | |
| --- | --- | --- | --- | --- |
| Cerebellar-Brainstem ROI | Limbic ROI | r | p | p_corr_ |
| Anterior Left | Angular Left | -0.018 | .830 | .918 |
| Anterior Left | Angular Right | 0.122 | .150 | .428 |
| Anterior Left | Cuneus Left | -0.072 | .396 | .630 |
| Anterior Left | Cuneus Right | -0.032 | .704 | .840 |
| Anterior Left | Fusiform Gyrus Left | 0.032 | .708 | .840 |
| Anterior Left | Fusiform Gyrus Right | 0.078 | .360 | .594 |
| Anterior Left | Inferior Frontal (opercular) Left | 0.187 | .027 | .244† |
| Anterior Left | Inferior Frontal (opercular) Right | 0.097 | .255 | .486 |
| Anterior Left | Inferior Frontal (triangular) Left | 0.120 | .157 | .428 |
| Anterior Left | Inferior Frontal (triangular) Right | 0.141 | .096 | .388 |
| Anterior Left | Inferior Orbital Left | 0.047 | .578 | .760 |
| Anterior Left | Inferior Orbital Right | 0.123 | .148 | .428 |
| Anterior Left | Inferior Parietal Left | -0.191 | .024 | .244† |
| Anterior Left | Inferior Parietal Right | 0.080 | .349 | .588 |
| Anterior Left | Inferior Temporal Left | 0.069 | .420 | .652 |
| Anterior Left | Inferior Temporal Right | 0.100 | .242 | .484 |
| Anterior Left | Lingual Left | 0.145 | .087 | .378 |
| Anterior Left | Lingual Right | 0.067 | .434 | .659 |
| Anterior Left | Middle Frontal Left | 0.023 | .787 | .888 |
| Anterior Left | Middle Frontal Right | 0.070 | .408 | .641 |
| Anterior Left | Middle Occipital Left | 0.088 | .304 | .539 |
| Anterior Left | Middle Occipital Right | -0.016 | .851 | .930 |
| Anterior Left | Middle Temporal Left | -0.092 | .280 | .520 |
| Anterior Left | Middle Temporal Right | 0.019 | .825 | .918 |
| Anterior Left | Paracentral Lobule Left | 0.193 | .022 | .244† |
| Anterior Left | Paracentral Lobule Right | 0.131 | .122 | .408 |
| Anterior Left | Precuneus Left | 0.150 | .076 | .347 |
| Anterior Left | Precuneus Right | 0.107 | .210 | .462 |
| Anterior Left | Rolandic Left | 0.132 | .121 | .408 |
| Anterior Left | Rolandic Right | 0.062 | .466 | .683 |
| Anterior Left | Superior Frontal (dorsal) Left | 0.076 | .375 | .609 |
| Anterior Left | Superior Frontal (dorsal) Right | 0.033 | .695 | .837 |
| Anterior Left | Superior Frontal (medial) Left | 0.144 | .090 | .379 |
| Anterior Left | Superior Frontal (medial) Right | 0.098 | .247 | .486 |
| Anterior Left | Supplemental Motor Area Left | 0.191 | .024 | .244† |
| Anterior Left | Supplemental Motor Area Right | 0.104 | .221 | .466 |
| Anterior Left | Supramarginal Left | 0.018 | .831 | .918 |
| Anterior Left | Supramarginal Right | -0.017 | .841 | .925 |
| Anterior Left | Superior Occipital Left | -0.064 | .453 | .678 |
| Anterior Left | Superior Occipital Right | 0.029 | .733 | .852 |
| Anterior Left | Superior Parietal Left | 0.036 | .676 | .824 |
| Anterior Left | Superior Parietal Right | 0.029 | .731 | .852 |
| Anterior Left | Superior Temporal Left | 0.025 | .771 | .881 |
| Anterior Left | Superior Temporal Right | 0.070 | .412 | .643 |
| Anterior Right | Angular Left | 0.020 | .813 | .910 |
| Anterior Right | Angular Right | 0.172 | .042 | .272† |
| Anterior Right | Cuneus Left | -0.040 | .636 | .807 |
| Anterior Right | Cuneus Right | -0.010 | .909 | .941 |
| Anterior Right | Fusiform Gyrus Left | 0.123 | .148 | .428 |
| Anterior Right | Fusiform Gyrus Right | 0.088 | .299 | .537 |
| Anterior Right | Inferior Frontal (opercular) Left | 0.178 | .036 | .256† |
| Anterior Right | Inferior Frontal (opercular) Right | 0.119 | .160 | .428 |
| Anterior Right | Inferior Frontal (triangular) Left | 0.059 | .491 | .695 |
| Anterior Right | Inferior Frontal (triangular) Right | 0.116 | .172 | .435 |
| Anterior Right | Inferior Orbital Left | 0.058 | .499 | .698 |
| Anterior Right | Inferior Orbital Right | 0.060 | .481 | .686 |
| Anterior Right | Inferior Parietal Left | -0.062 | .470 | .684 |
| Anterior Right | Inferior Parietal Right | 0.129 | .129 | .419 |
| Anterior Right | Inferior Temporal Left | 0.063 | .457 | .678 |
| Anterior Right | Inferior Temporal Right | 0.109 | .202 | .448 |
| Anterior Right | Lingual Left | 0.097 | .253 | .486 |
| Anterior Right | Lingual Right | 0.085 | .317 | .555 |
| Anterior Right | Middle Frontal Left | -0.012 | .887 | .932 |
| Anterior Right | Middle Frontal Right | 0.113 | .183 | .444 |
| Anterior Right | Middle Occipital Left | -0.013 | .881 | .932 |
| Anterior Right | Middle Occipital Right | -0.030 | .725 | .851 |
| Anterior Right | Middle Temporal Left | -0.122 | .151 | .428 |
| Anterior Right | Middle Temporal Right | 0.041 | .633 | .807 |
| Anterior Right | Paracentral Lobule Left | 0.143 | .092 | .380 |
| Anterior Right | Paracentral Lobule Right | 0.110 | .196 | .448 |
| Anterior Right | Precuneus Left | 0.173 | .041 | .272† |
| Anterior Right | Precuneus Right | 0.087 | .304 | .539 |
| Anterior Right | Rolandic Left | 0.041 | .629 | .806 |
| Anterior Right | Rolandic Right | 0.031 | .713 | .841 |
| Anterior Right | Superior Frontal (dorsal) Left | 0.048 | .574 | .757 |
| Anterior Right | Superior Frontal (dorsal) Right | 0.076 | .374 | .609 |
| Anterior Right | Superior Frontal (medial) Left | 0.119 | .160 | .428 |
| Anterior Right | Superior Frontal (medial) Right | 0.064 | .452 | .678 |
| Anterior Right | Supplemental Motor Area Left | 0.168 | .047 | .291† |
| Anterior Right | Supplemental Motor Area Right | 0.121 | .153 | .428 |
| Anterior Right | Supramarginal Left | -0.013 | .876 | .932 |
| Anterior Right | Supramarginal Right | -0.048 | .574 | .757 |
| Anterior Right | Superior Occipital Left | -0.132 | .119 | .408 |
| Anterior Right | Superior Occipital Right | 0.013 | .875 | .932 |
| Anterior Right | Superior Parietal Left | 0.082 | .337 | .577 |
| Anterior Right | Superior Parietal Right | 0.113 | .185 | .445 |
| Anterior Right | Superior Temporal Left | -0.024 | .774 | .881 |
| Anterior Right | Superior Temporal Right | 0.044 | .606 | .784 |
| Brainstem | Angular Left | 0.079 | .352 | .588 |
| Brainstem | Angular Right | 0.255 | .002 | .178† |
| Brainstem | Cuneus Left | -0.022 | .800 | .898 |
| Brainstem | Cuneus Right | -0.099 | .244 | .484 |
| Brainstem | Fusiform Gyrus Left | 0.190 | .024 | .244† |
| Brainstem | Fusiform Gyrus Right | 0.164 | .054 | .307 |
| Brainstem | Inferior Frontal (opercular) Left | 0.263 | .002 | .178† |
| Brainstem | Inferior Frontal (opercular) Right | 0.146 | .085 | .374 |
| Brainstem | Inferior Frontal (triangular) Left | 0.181 | .032 | .244† |
| Brainstem | Inferior Frontal (triangular) Right | 0.123 | .146 | .428 |
| Brainstem | Inferior Orbital Left | 0.079 | .352 | .588 |
| Brainstem | Inferior Orbital Right | 0.130 | .127 | .419 |
| Brainstem | Inferior Parietal Left | 0.010 | .908 | .941 |
| Brainstem | Inferior Parietal Right | 0.196 | .020 | .244† |
| Brainstem | Inferior Temporal Left | 0.039 | .647 | .811 |
| Brainstem | Inferior Temporal Right | 0.105 | .215 | .466 |
| Brainstem | Lingual Left | 0.090 | .293 | .533 |
| Brainstem | Lingual Right | 0.104 | .222 | .466 |
| Brainstem | Middle Frontal Left | 0.150 | .076 | .347 |
| Brainstem | Middle Frontal Right | 0.149 | .080 | .356 |
| Brainstem | Middle Occipital Left | 0.039 | .649 | .811 |
| Brainstem | Middle Occipital Right | 0.005 | .950 | .958 |
| Brainstem | Middle Temporal Left | 0.094 | .271 | .507 |
| Brainstem | Middle Temporal Right | 0.068 | .426 | .658 |
| Brainstem | Paracentral Lobule Left | 0.183 | .030 | .244† |
| Brainstem | Paracentral Lobule Right | 0.109 | .202 | .448 |
| Brainstem | Precuneus Left | 0.193 | .022 | .244† |
| Brainstem | Precuneus Right | 0.186 | .028 | .244† |
| Brainstem | Rolandic Left | 0.108 | .202 | .448 |
| Brainstem | Rolandic Right | 0.014 | .870 | .932 |
| Brainstem | Superior Frontal (dorsal) Left | 0.151 | .075 | .347 |
| Brainstem | Superior Frontal (dorsal) Right | 0.119 | .160 | .428 |
| Brainstem | Superior Frontal (medial) Left | 0.216 | .010 | .244† |
| Brainstem | Superior Frontal (medial) Right | 0.135 | .111 | .399 |
| Brainstem | Supplemental Motor Area Left | 0.219 | .009 | .244† |
| Brainstem | Supplemental Motor Area Right | 0.188 | .026 | .244† |
| Brainstem | Supramarginal Left | 0.053 | .534 | .734 |
| Brainstem | Supramarginal Right | 0.097 | .255 | .486 |
| Brainstem | Superior Occipital Left | -0.074 | .387 | .619 |
| Brainstem | Superior Occipital Right | -0.051 | .553 | .748 |
| Brainstem | Superior Parietal Left | 0.099 | .244 | .484 |
| Brainstem | Superior Parietal Right | 0.174 | .040 | .272† |
| Brainstem | Superior Temporal Left | 0.104 | .220 | .466 |
| Brainstem | Superior Temporal Right | 0.095 | .267 | .503 |
| Posterior Left | Angular Left | 0.039 | .645 | .811 |
| Posterior Left | Angular Right | 0.166 | .050 | .296† |
| Posterior Left | Cuneus Left | -0.003 | .968 | .971 |
| Posterior Left | Cuneus Right | -0.009 | .916 | .943 |
| Posterior Left | Fusiform Gyrus Left | -0.013 | .883 | .932 |
| Posterior Left | Fusiform Gyrus Right | 0.061 | .472 | .684 |
| Posterior Left | Inferior Frontal (opercular) Left | 0.216 | .011 | .244† |
| Posterior Left | Inferior Frontal (opercular) Right | 0.248 | .003 | .178† |
| Posterior Left | Inferior Frontal (triangular) Left | 0.151 | .076 | .347 |
| Posterior Left | Inferior Frontal (triangular) Right | 0.223 | .008 | .244† |
| Posterior Left | Inferior Orbital Left | -0.075 | .381 | .614 |
| Posterior Left | Inferior Orbital Right | 0.058 | .492 | .695 |
| Posterior Left | Inferior Parietal Left | -0.037 | .664 | .823 |
| Posterior Left | Inferior Parietal Right | 0.110 | .195 | .448 |
| Posterior Left | Inferior Temporal Left | -0.051 | .548 | .746 |
| Posterior Left | Inferior Temporal Right | 0.032 | .710 | .840 |
| Posterior Left | Lingual Left | 0.123 | .148 | .428 |
| Posterior Left | Lingual Right | 0.178 | .035 | .256† |
| Posterior Left | Middle Frontal Left | 0.159 | .060 | .333 |
| Posterior Left | Middle Frontal Right | 0.197 | .020 | .244† |
| Posterior Left | Middle Occipital Left | 0.052 | .539 | .737 |
| Posterior Left | Middle Occipital Right | 0.028 | .741 | .855 |
| Posterior Left | Middle Temporal Left | -0.047 | .583 | .762 |
| Posterior Left | Middle Temporal Right | 0.071 | .404 | .638 |
| Posterior Left | Paracentral Lobule Left | 0.154 | .070 | .347 |
| Posterior Left | Paracentral Lobule Right | 0.125 | .141 | .428 |
| Posterior Left | Precuneus Left | 0.123 | .147 | .428 |
| Posterior Left | Precuneus Right | 0.120 | .158 | .428 |
| Posterior Left | Rolandic Left | 0.086 | .313 | .550 |
| Posterior Left | Rolandic Right | 0.012 | .890 | .932 |
| Posterior Left | Superior Frontal (dorsal) Left | 0.183 | .030 | .244† |
| Posterior Left | Superior Frontal (dorsal) Right | 0.207 | .014 | .244† |
| Posterior Left | Superior Frontal (medial) Left | 0.135 | .112 | .399 |
| Posterior Left | Superior Frontal (medial) Right | 0.157 | .063 | .333 |
| Posterior Left | Supplemental Motor Area Left | 0.205 | .015 | .244† |
| Posterior Left | Supplemental Motor Area Right | 0.226 | .007 | .244† |
| Posterior Left | Supramarginal Left | 0.117 | .168 | .435 |
| Posterior Left | Supramarginal Right | 0.210 | .013 | .244† |
| Posterior Left | Superior Occipital Left | 0.007 | .937 | .957 |
| Posterior Left | Superior Occipital Right | 0.009 | .918 | .943 |
| Posterior Left | Superior Parietal Left | 0.067 | .432 | .659 |
| Posterior Left | Superior Parietal Right | 0.154 | .070 | .347 |
| Posterior Left | Superior Temporal Left | 0.037 | .668 | .824 |
| Posterior Left | Superior Temporal Right | 0.049 | .565 | .757 |
| Posterior Right | Angular Left | 0.135 | .111 | .399 |
| Posterior Right | Angular Right | 0.144 | .090 | .379 |
| Posterior Right | Cuneus Left | 0.007 | .939 | .957 |
| Posterior Right | Cuneus Right | 0.042 | .620 | .798 |
| Posterior Right | Fusiform Gyrus Left | 0.182 | .031 | .244† |
| Posterior Right | Fusiform Gyrus Right | 0.109 | .200 | .448 |
| Posterior Right | Inferior Frontal (opercular) Left | 0.182 | .031 | .244† |
| Posterior Right | Inferior Frontal (opercular) Right | 0.169 | .046 | .291† |
| Posterior Right | Inferior Frontal (triangular) Left | 0.106 | .214 | .466 |
| Posterior Right | Inferior Frontal (triangular) Right | 0.109 | .198 | .448 |
| Posterior Right | Inferior Orbital Left | 0.102 | .233 | .484 |
| Posterior Right | Inferior Orbital Right | 0.050 | .555 | .748 |
| Posterior Right | Inferior Parietal Left | 0.136 | .109 | .399 |
| Posterior Right | Inferior Parietal Right | 0.075 | .376 | .609 |
| Posterior Right | Inferior Temporal Left | 0.028 | .739 | .855 |
| Posterior Right | Inferior Temporal Right | 0.013 | .877 | .932 |
| Posterior Right | Lingual Left | 0.115 | .175 | .439 |
| Posterior Right | Lingual Right | 0.117 | .169 | .435 |
| Posterior Right | Middle Frontal Left | 0.132 | .120 | .408 |
| Posterior Right | Middle Frontal Right | 0.113 | .183 | .444 |
| Posterior Right | Middle Occipital Left | 0.100 | .241 | .484 |
| Posterior Right | Middle Occipital Right | -0.114 | .178 | .444 |
| Posterior Right | Middle Temporal Left | -0.032 | .705 | .840 |
| Posterior Right | Middle Temporal Right | 0.044 | .605 | .784 |
| Posterior Right | Paracentral Lobule Left | 0.132 | .121 | .408 |
| Posterior Right | Paracentral Lobule Right | 0.089 | .295 | .534 |
| Posterior Right | Precuneus Left | 0.138 | .103 | .399 |
| Posterior Right | Precuneus Right | 0.172 | .042 | .272† |
| Posterior Right | Rolandic Left | 0.063 | .463 | .682 |
| Posterior Right | Rolandic Right | -0.024 | .782 | .886 |
| Posterior Right | Superior Frontal (dorsal) Left | 0.153 | .072 | .347 |
| Posterior Right | Superior Frontal (dorsal) Right | 0.104 | .222 | .466 |
| Posterior Right | Superior Frontal (medial) Left | 0.195 | .021 | .244† |
| Posterior Right | Superior Frontal (medial) Right | 0.100 | .238 | .484 |
| Posterior Right | Supplemental Motor Area Left | 0.246 | .003 | .178† |
| Posterior Right | Supplemental Motor Area Right | 0.210 | .013 | .244† |
| Posterior Right | Supramarginal Left | 0.097 | .256 | .486 |
| Posterior Right | Supramarginal Right | 0.157 | .064 | .333 |
| Posterior Right | Superior Occipital Left | 0.060 | .480 | .686 |
| Posterior Right | Superior Occipital Right | -0.038 | .652 | .812 |
| Posterior Right | Superior Parietal Left | 0.067 | .433 | .659 |
| Posterior Right | Superior Parietal Right | 0.120 | .159 | .428 |
| Posterior Right | Superior Temporal Left | 0.002 | .979 | .979 |
| Posterior Right | Superior Temporal Right | 0.025 | .770 | .881 |
| Vermis | Angular Left | 0.036 | .676 | .824 |
| Vermis | Angular Right | 0.181 | .032 | .244† |
| Vermis | Cuneus Left | -0.058 | .499 | .698 |
| Vermis | Cuneus Right | -0.064 | .455 | .678 |
| Vermis | Fusiform Gyrus Left | 0.118 | .163 | .431 |
| Vermis | Fusiform Gyrus Right | 0.135 | .112 | .399 |
| Vermis | Inferior Frontal (opercular) Left | 0.166 | .050 | .296 |
| Vermis | Inferior Frontal (opercular) Right | 0.109 | .199 | .448 |
| Vermis | Inferior Frontal (triangular) Left | 0.060 | .478 | .686 |
| Vermis | Inferior Frontal (triangular) Right | 0.055 | .522 | .725 |
| Vermis | Inferior Orbital Left | 0.034 | .686 | .831 |
| Vermis | Inferior Orbital Right | 0.079 | .355 | .589 |
| Vermis | Inferior Parietal Left | -0.005 | .951 | .958 |
| Vermis | Inferior Parietal Right | 0.139 | .102 | .399 |
| Vermis | Inferior Temporal Left | -0.012 | .888 | .932 |
| Vermis | Inferior Temporal Right | 0.048 | .573 | .757 |
| Vermis | Lingual Left | 0.139 | .102 | .399 |
| Vermis | Lingual Right | 0.138 | .104 | .399 |
| Vermis | Middle Frontal Left | 0.053 | .533 | .734 |
| Vermis | Middle Frontal Right | 0.158 | .063 | .333 |
| Vermis | Middle Occipital Left | 0.113 | .183 | .444 |
| Vermis | Middle Occipital Right | 0.012 | .885 | .932 |
| Vermis | Middle Temporal Left | 0.005 | .950 | .958 |
| Vermis | Middle Temporal Right | 0.092 | .282 | .520 |
| Vermis | Paracentral Lobule Left | 0.270 | .001 | .178† |
| Vermis | Paracentral Lobule Right | 0.208 | .014 | .244† |
| Vermis | Precuneus Left | 0.195 | .021 | .244† |
| Vermis | Precuneus Right | 0.237 | .005 | .208† |
| Vermis | Rolandic Left | 0.083 | .332 | .573 |
| Vermis | Rolandic Right | -0.010 | .904 | .941 |
| Vermis | Superior Frontal (dorsal) Left | 0.100 | .239 | .484 |
| Vermis | Superior Frontal (dorsal) Right | 0.110 | .196 | .448 |
| Vermis | Superior Frontal (medial) Left | 0.125 | .140 | .428 |
| Vermis | Superior Frontal (medial) Right | 0.127 | .136 | .428 |
| Vermis | Supplemental Motor Area Left | 0.196 | .020 | .244† |
| Vermis | Supplemental Motor Area Right | 0.157 | .063 | .333 |
| Vermis | Supramarginal Left | 0.090 | .291 | .533 |
| Vermis | Supramarginal Right | 0.116 | .171 | .435 |
| Vermis | Superior Occipital Left | 0.016 | .853 | .930 |
| Vermis | Superior Occipital Right | -0.035 | .678 | .824 |
| Vermis | Superior Parietal Left | 0.098 | .249 | .486 |
| Vermis | Superior Parietal Right | 0.122 | .151 | .428 |
| Vermis | Superior Temporal Left | 0.080 | .346 | .588 |
| Vermis | Superior Temporal Right | 0.083 | .328 | .570 |
| Full correlation table for cerebellar-brainstem and paralimbic ROIs. Significant connections are highlighted yellow if they survive FDR correction and blue if they were significant prior to correction but not after | | | | |

| Supplemental Information 7: All Cerebellar-Brainstem and Paralimbic ROI Connections | | | | |
| --- | --- | --- | --- | --- |
| Cerebellar-Brainstem ROI | Paralimbic ROI | r | p | p_corr_ |
| Anterior Left | Anterior Cingulate Left | 0.190 | .025 | .154† |
| Anterior Left | Anterior Cingulate Right | 0.207 | .014 | .125† |
| Anterior Left | Insula Left | 0.018 | .830 | .885 |
| Anterior Left | Insula Right | 0.148 | .081 | .237 |
| Anterior Left | Middle Cingulate Left | 0.247 | .003 | .064† |
| Anterior Left | Middle Cingulate Right | 0.200 | .018 | .129† |
| Anterior Left | Orbital (Inferior) Left | 0.089 | .294 | .437 |
| Anterior Left | Orbital (Inferior) Right | 0.140 | .100 | .245 |
| Anterior Left | Orbital (Medial) Left | 0.064 | .456 | .568 |
| Anterior Left | Orbital (Medial) Right | 0.034 | .688 | .768 |
| Anterior Left | Orbital (Middle) Left | 0.048 | .569 | .656 |
| Anterior Left | Orbital (Middle) Right | -0.021 | .807 | .881 |
| Anterior Left | Orbital (Superior) Left | 0.151 | .075 | .226 |
| Anterior Left | Orbital (Superior) Right | -0.060 | .481 | .587 |
| Anterior Left | Parahippocamus Left | 0.122 | .150 | .304 |
| Anterior Left | Parahippocamus Right | 0.125 | .143 | .302 |
| Anterior Left | Posterior Cingulate Left | 0.036 | .677 | .761 |
| Anterior Left | Posterior Cingulate Right | 0.095 | .265 | .410 |
| Anterior Left | Rectus Left | 0.040 | .643 | .729 |
| Anterior Left | Rectus Right | -0.049 | .568 | .656 |
| Anterior Left | Temporal Pole (Middle) Left | -0.063 | .458 | .568 |
| Anterior Left | Temporal Pole (Middle) Right | -0.114 | .180 | .324 |
| Anterior Left | Temporal Pole (Superior) Left | 0.066 | .438 | .562 |
| Anterior Left | Temporal Pole (Superior) Right | 0.098 | .251 | .402 |
| Anterior Right | Anterior Cingulate Left | 0.197 | .020 | .129† |
| Anterior Right | Anterior Cingulate Right | 0.156 | .066 | .218 |
| Anterior Right | Insula Left | -0.063 | .462 | .568 |
| Anterior Right | Insula Right | 0.115 | .175 | .324 |
| Anterior Right | Middle Cingulate Left | 0.202 | .017 | .129† |
| Anterior Right | Middle Cingulate Right | 0.178 | .035 | .174† |
| Anterior Right | Orbital (Inferior) Left | 0.113 | .184 | .326 |
| Anterior Right | Orbital (Inferior) Right | 0.108 | .205 | .348 |
| Anterior Right | Orbital (Medial) Left | 0.105 | .218 | .357 |
| Anterior Right | Orbital (Medial) Right | 0.078 | .358 | .508 |
| Anterior Right | Orbital (Middle) Left | 0.049 | .569 | .656 |
| Anterior Right | Orbital (Middle) Right | 0.017 | .844 | .893 |
| Anterior Right | Orbital (Superior) Left | 0.179 | .034 | .174† |
| Anterior Right | Orbital (Superior) Right | -0.011 | .896 | .922 |
| Anterior Right | Parahippocamus Left | 0.137 | .107 | .245 |
| Anterior Right | Parahippocamus Right | 0.113 | .186 | .326 |
| Anterior Right | Posterior Cingulate Left | 0.052 | .544 | .647 |
| Anterior Right | Posterior Cingulate Right | 0.151 | .075 | .226 |
| Anterior Right | Rectus Left | 0.136 | .110 | .248 |
| Anterior Right | Rectus Right | 0.077 | .364 | .508 |
| Anterior Right | Temporal Pole (Middle) Left | -0.015 | .863 | .900 |
| Anterior Right | Temporal Pole (Middle) Right | -0.051 | .549 | .649 |
| Anterior Right | Temporal Pole (Superior) Left | 0.056 | .508 | .615 |
| Anterior Right | Temporal Pole (Superior) Right | 0.081 | .343 | .499 |
| Brainstem | Anterior Cingulate Left | 0.257 | .002 | .064† |
| Brainstem | Anterior Cingulate Right | 0.173 | .041 | .189† |
| Brainstem | Insula Left | 0.094 | .270 | .410 |
| Brainstem | Insula Right | 0.116 | .174 | .324 |
| Brainstem | Middle Cingulate Left | 0.208 | .014 | .125† |
| Brainstem | Middle Cingulate Right | 0.221 | .009 | .125† |
| Brainstem | Orbital (Inferior) Left | 0.123 | .149 | .304 |
| Brainstem | Orbital (Inferior) Right | 0.163 | .054 | .199 |
| Brainstem | Orbital (Medial) Left | 0.114 | .178 | .324 |
| Brainstem | Orbital (Medial) Right | 0.081 | .340 | .499 |
| Brainstem | Orbital (Middle) Left | 0.078 | .360 | .508 |
| Brainstem | Orbital (Middle) Right | 0.118 | .167 | .324 |
| Brainstem | Orbital (Superior) Left | 0.168 | .048 | .189† |
| Brainstem | Orbital (Superior) Right | 0.078 | .358 | .508 |
| Brainstem | Parahippocamus Left | 0.137 | .107 | .245 |
| Brainstem | Parahippocamus Right | 0.068 | .422 | .553 |
| Brainstem | Posterior Cingulate Left | 0.137 | .107 | .245 |
| Brainstem | Posterior Cingulate Right | 0.116 | .172 | .324 |
| Brainstem | Rectus Left | 0.198 | .019 | .129† |
| Brainstem | Rectus Right | 0.166 | .050 | .189† |
| Brainstem | Temporal Pole (Middle) Left | 0.096 | .259 | .409 |
| Brainstem | Temporal Pole (Middle) Right | -0.042 | .626 | .716 |
| Brainstem | Temporal Pole (Superior) Left | 0.172 | .043 | .189† |
| Brainstem | Temporal Pole (Superior) Right | 0.123 | .149 | .304 |
| Posterior Left | Anterior Cingulate Left | 0.153 | .072 | .225 |
| Posterior Left | Anterior Cingulate Right | 0.144 | .089 | .241 |
| Posterior Left | Insula Left | 0.094 | .268 | .410 |
| Posterior Left | Insula Right | 0.074 | .383 | .525 |
| Posterior Left | Middle Cingulate Left | 0.155 | .067 | .218 |
| Posterior Left | Middle Cingulate Right | 0.181 | .032 | .172† |
| Posterior Left | Orbital (Inferior) Left | 0.112 | .189 | .328 |
| Posterior Left | Orbital (Inferior) Right | 0.155 | .068 | .218 |
| Posterior Left | Orbital (Medial) Left | 0.247 | .003 | .064† |
| Posterior Left | Orbital (Medial) Right | 0.182 | .031 | .172† |
| Posterior Left | Orbital (Middle) Left | 0.027 | .753 | .827 |
| Posterior Left | Orbital (Middle) Right | 0.053 | .535 | .642 |
| Posterior Left | Orbital (Superior) Left | 0.245 | .004 | .064† |
| Posterior Left | Orbital (Superior) Right | 0.198 | .019 | .129† |
| Posterior Left | Parahippocamus Left | 0.105 | .218 | .357 |
| Posterior Left | Parahippocamus Right | 0.072 | .399 | .535 |
| Posterior Left | Posterior Cingulate Left | 0.171 | .043 | .189† |
| Posterior Left | Posterior Cingulate Right | 0.198 | .019 | .129† |
| Posterior Left | Rectus Left | 0.267 | .001 | .064† |
| Posterior Left | Rectus Right | 0.220 | .009 | .125† |
| Posterior Left | Temporal Pole (Middle) Left | 0.007 | .931 | .944 |
| Posterior Left | Temporal Pole (Middle) Right | -0.002 | .978 | .978 |
| Posterior Left | Temporal Pole (Superior) Left | 0.115 | .178 | .324 |
| Posterior Left | Temporal Pole (Superior) Right | 0.142 | .095 | .245 |
| Posterior Right | Anterior Cingulate Left | 0.138 | .105 | .245 |
| Posterior Right | Anterior Cingulate Right | 0.146 | .086 | .241 |
| Posterior Right | Insula Left | 0.072 | .401 | .535 |
| Posterior Right | Insula Right | 0.063 | .460 | .568 |
| Posterior Right | Middle Cingulate Left | 0.155 | .067 | .218 |
| Posterior Right | Middle Cingulate Right | 0.209 | .013 | .125† |
| Posterior Right | Orbital (Inferior) Left | 0.141 | .097 | .245 |
| Posterior Right | Orbital (Inferior) Right | 0.159 | .061 | .215 |
| Posterior Right | Orbital (Medial) Left | 0.066 | .439 | .562 |
| Posterior Right | Orbital (Medial) Right | 0.033 | .700 | .775 |
| Posterior Right | Orbital (Middle) Left | -0.006 | .943 | .949 |
| Posterior Right | Orbital (Middle) Right | 0.009 | .913 | .932 |
| Posterior Right | Orbital (Superior) Left | 0.121 | .153 | .306 |
| Posterior Right | Orbital (Superior) Right | 0.104 | .221 | .358 |
| Posterior Right | Parahippocamus Left | 0.095 | .262 | .410 |
| Posterior Right | Parahippocamus Right | 0.109 | .199 | .340 |
| Posterior Right | Posterior Cingulate Left | 0.210 | .013 | .125† |
| Posterior Right | Posterior Cingulate Right | 0.253 | .003 | .064† |
| Posterior Right | Rectus Left | 0.139 | .102 | .245 |
| Posterior Right | Rectus Right | 0.167 | .049 | .189† |
| Posterior Right | Temporal Pole (Middle) Left | 0.019 | .822 | .885 |
| Posterior Right | Temporal Pole (Middle) Right | -0.126 | .137 | .299 |
| Posterior Right | Temporal Pole (Superior) Left | 0.126 | .139 | .299 |
| Posterior Right | Temporal Pole (Superior) Right | 0.070 | .413 | .545 |
| Vermis | Anterior Cingulate Left | 0.144 | .091 | .241 |
| Vermis | Anterior Cingulate Right | 0.184 | .029 | .172† |
| Vermis | Insula Left | 0.019 | .824 | .885 |
| Vermis | Insula Right | 0.075 | .381 | .525 |
| Vermis | Middle Cingulate Left | 0.218 | .010 | .125† |
| Vermis | Middle Cingulate Right | 0.183 | .030 | .172† |
| Vermis | Orbital (Inferior) Left | 0.144 | .090 | .241 |
| Vermis | Orbital (Inferior) Right | 0.162 | .057 | .204 |
| Vermis | Orbital (Medial) Left | 0.132 | .121 | .268 |
| Vermis | Orbital (Medial) Right | 0.089 | .294 | .437 |
| Vermis | Orbital (Middle) Left | 0.119 | .160 | .316 |
| Vermis | Orbital (Middle) Right | 0.073 | .394 | .535 |
| Vermis | Orbital (Superior) Left | 0.260 | .002 | .064† |
| Vermis | Orbital (Superior) Right | 0.066 | .441 | .562 |
| Vermis | Parahippocamus Left | 0.166 | .049 | .189† |
| Vermis | Parahippocamus Right | 0.147 | .084 | .241 |
| Vermis | Posterior Cingulate Left | 0.137 | .106 | .245 |
| Vermis | Posterior Cingulate Right | 0.284 | .001 | .064† |
| Vermis | Rectus Left | 0.215 | .011 | .125† |
| Vermis | Rectus Right | 0.166 | .050 | .189† |
| Vermis | Temporal Pole (Middle) Left | 0.016 | .855 | .898 |
| Vermis | Temporal Pole (Middle) Right | 0.011 | .894 | .922 |
| Vermis | Temporal Pole (Superior) Left | 0.106 | .214 | .357 |
| Vermis | Temporal Pole (Superior) Right | 0.166 | .050 | .189† |
| Full correlation table for cerebellar-brainstem and paralimbic ROIs. Significant connections are highlighted yellow if they survive FDR correction and blue if they were significant prior to correction but not after | | | | |

| Supplemental Information 8: All Cerebellar-Brainstem and Limbic ROI Connections | | | | |
| --- | --- | --- | --- | --- |
| Cerebellar-Brainstem ROI | Limbic ROI | r | p | p_corr_ |
| Anterior Left | Amygdala Left | 0.139 | .103 | .223 |
| Anterior Left | Amygdala Right | 0.104 | .221 | .306 |
| Anterior Left | Hippocampus Left | 0.148 | .080 | .218 |
| Anterior Left | Hippocampus Right | 0.084 | .323 | .376 |
| Anterior Left | Olfactory Left | 0.027 | .748 | .748 |
| Anterior Left | Olfactory Right | 0.061 | .474 | .502 |
| Anterior Right | Amygdala Left | 0.057 | .504 | .518 |
| Anterior Right | Amygdala Right | 0.121 | .155 | .233 |
| Anterior Right | Hippocampus Left | 0.098 | .250 | .309 |
| Anterior Right | Hippocampus Right | 0.098 | .251 | .309 |
| Anterior Right | Olfactory Left | 0.137 | .106 | .223 |
| Anterior Right | Olfactory Right | 0.177 | .037 | .146† |
| Brainstem | Amygdala Left | 0.096 | .257 | .309 |
| Brainstem | Amygdala Right | 0.064 | .449 | .490 |
| Brainstem | Hippocampus Left | 0.161 | .057 | .201 |
| Brainstem | Hippocampus Right | 0.077 | .366 | .411 |
| Brainstem | Olfactory Left | 0.101 | .235 | .309 |
| Brainstem | Olfactory Right | 0.197 | .020 | .119† |
| Posterior Left | Amygdala Left | 0.205 | .015 | .119† |
| Posterior Left | Amygdala Right | 0.133 | .117 | .223 |
| Posterior Left | Hippocampus Left | 0.135 | .113 | .223 |
| Posterior Left | Hippocampus Right | 0.106 | .214 | .306 |
| Posterior Left | Olfactory Left | 0.200 | .018 | .119† |
| Posterior Left | Olfactory Right | 0.198 | .019 | .119† |
| Posterior Right | Amygdala Left | 0.133 | .119 | .223 |
| Posterior Right | Amygdala Right | 0.125 | .142 | .223 |
| Posterior Right | Hippocampus Left | 0.157 | .064 | .201 |
| Posterior Right | Hippocampus Right | 0.126 | .139 | .223 |
| Posterior Right | Olfactory Left | 0.155 | .067 | .201 |
| Posterior Right | Olfactory Right | 0.224 | .008 | .119† |
| Vermis | Amygdala Left | 0.146 | .085 | .218 |
| Vermis | Amygdala Right | 0.128 | .132 | .223 |
| Vermis | Hippocampus Left | 0.125 | .141 | .223 |
| Vermis | Hippocampus Right | 0.181 | .032 | .145† |
| Vermis | Olfactory Left | 0.182 | .031 | .145† |
| Vermis | Olfactory Right | 0.210 | .013 | .119† |
| Full correlation table for cerebellar-brainstem and paralimbic ROIs. Significant connections are highlighted yellow if they survive FDR correction and blue if they were significant prior to correction but not after | | | | |

| Supplemental Information 9: All Subcortical and Limbic ROI Connections | | | | |
| --- | --- | --- | --- | --- |
| Cerebellar-Brainstem ROI | Limbic ROI | r | p | p_corr_ |
| Caudate Left | Amygdala Left | 0.136 | .110 | .277 |
| Caudate Left | Amygdala Right | 0.083 | .329 | .415 |
| Caudate Left | Hippocampus Left | 0.056 | .510 | .544 |
| Caudate Left | Hippocampus Right | 0.117 | .170 | .327 |
| Caudate Left | Olfactory Left | 0.136 | .109 | .277 |
| Caudate Left | Olfactory Right | 0.170 | .045 | .228† |
| Caudate Right | Amygdala Left | 0.137 | .106 | .277 |
| Caudate Right | Amygdala Right | 0.101 | .234 | .367 |
| Caudate Right | Hippocampus Left | 0.187 | .027 | .184† |
| Caudate Right | Hippocampus Right | 0.088 | .299 | .388 |
| Caudate Right | Olfactory Left | 0.242 | .004 | .129† |
| Caudate Right | Olfactory Right | 0.213 | .012 | .162† |
| Palladium Left | Amygdala Left | 0.168 | .048 | .228† |
| Palladium Left | Amygdala Right | 0.140 | .098 | .277 |
| Palladium Left | Hippocampus Left | 0.146 | .085 | .277 |
| Palladium Left | Hippocampus Right | 0.077 | .364 | .448 |
| Palladium Left | Olfactory Left | 0.208 | .013 | .162† |
| Palladium Left | Olfactory Right | 0.234 | .005 | .129† |
| Palladium Right | Amygdala Left | 0.128 | .132 | .289 |
| Palladium Right | Amygdala Right | 0.117 | .167 | .327 |
| Palladium Right | Hippocampus Left | 0.093 | .272 | .368 |
| Palladium Right | Hippocampus Right | 0.030 | .722 | .722 |
| Palladium Right | Olfactory Left | 0.201 | .018 | .168† |
| Palladium Right | Olfactory Right | 0.174 | .040 | .228† |
| Putamen Left | Amygdala Left | 0.129 | .130 | .289 |
| Putamen Left | Amygdala Right | 0.110 | .196 | .362 |
| Putamen Left | Hippocampus Left | 0.096 | .257 | .367 |
| Putamen Left | Hippocampus Right | 0.046 | .587 | .612 |
| Putamen Left | Olfactory Left | 0.157 | .064 | .277 |
| Putamen Left | Olfactory Right | 0.093 | .276 | .368 |
| Putamen Right | Amygdala Left | 0.064 | .449 | .490 |
| Putamen Right | Amygdala Right | 0.068 | .424 | .480 |
| Putamen Right | Hippocampus Left | 0.067 | .430 | .480 |
| Putamen Right | Hippocampus Right | 0.044 | .610 | .623 |
| Putamen Right | Olfactory Left | 0.129 | .129 | .289 |
| Putamen Right | Olfactory Right | 0.107 | .208 | .367 |
| Thalamus Left | Amygdala Left | 0.193 | .022 | .177† |
| Thalamus Left | Amygdala Right | 0.138 | .105 | .277 |
| Thalamus Left | Hippocampus Left | 0.153 | .071 | .277 |
| Thalamus Left | Hippocampus Right | 0.096 | .260 | .367 |
| Thalamus Left | Olfactory Left | 0.119 | .162 | .327 |
| Thalamus Left | Olfactory Right | 0.150 | .077 | .277 |
| Thalamus Right | Amygdala Left | 0.099 | .245 | .367 |
| Thalamus Right | Amygdala Right | 0.102 | .228 | .367 |
| Thalamus Right | Hippocampus Left | 0.074 | .387 | .453 |
| Thalamus Right | Hippocampus Right | 0.075 | .379 | .453 |
| Thalamus Right | Olfactory Left | 0.096 | .257 | .367 |
| Thalamus Right | Olfactory Right | 0.102 | .232 | .367 |
| Full correlation table for cerebellar-brainstem and paralimbic ROIs. Significant connections are highlighted yellow if they survive FDR correction and blue if they were significant prior to correction but not after | | | | |

Supplemental Information 10

When accounting for maternal sociodemographic factors on the relationship between skin breaks and neurodevelopment, the association remains durable for MCHAT scores. When accounting for maternal education, education was a significant predictor (F=10.135, p<.001), while skin breaks and the interaction between breaks and GA at birth remained significant (ps <.012), with posthoc analyses suggesting greater educational attainment is associated with lower MCHAT scores. Similarly for maternal race, race was a predictor (F=8.335, p<.001), with breaks and the interaction still significant (ps<.001). Finally, maternal employment status was not significant, while breaks and the interaction remained significant (ps <.046)

Conversely, none of the maternal sociodemographic factors were associated with gross (ps>.171) or fine (ps>.119) motor scales at 18-months while skin breaks remained significant (ps<.036). Finally, for expressed language, maternal race (F=4.414, p=.013), education (F=6.642, p=.001), and employment (F=7.059, p<0.001) were all significant predictors, while skin breaks (ps<.024) and the interactions with GA at birth remained significant (ps<.014).
